# Effects of Combined Oxytocin and Beta-3 Receptor Agonist (CL 316243) Treatment on Body Weight and Adiposity in Male Diet-Induced Obese Rats

**DOI:** 10.3389/fphys.2021.725912

**Published:** 2021-09-08

**Authors:** Melise M. Edwards, Ha K. Nguyen, Andrew D. Dodson, Adam J. Herbertson, Tomasz A. Wietecha, Tami Wolden-Hanson, James L. Graham, Mackenzie K. Honeycutt, Jared D. Slattery, Kevin D. O’Brien, Peter J. Havel, James E. Blevins

**Affiliations:** ^1^VA Puget Sound Health Care System, Office of Research and Development Medical Research Service, Department of Veterans Affairs Medical Center, Seattle, WA, United States; ^2^Division of Metabolism, Endocrinology and Nutrition, Department of Medicine, University of Washington School of Medicine, Seattle, WA, United States; ^3^UW Medicine Diabetes Institute, University of Washington School of Medicine, Seattle, WA, United States; ^4^Department of Nutrition, University of California, Davis, Davis, CA, United States; ^5^Division of Cardiology, Department of Medicine, University of Washington School of Medicine, Seattle, WA, United States; ^6^Department of Molecular Biosciences, School of Veterinary Medicine, University of California, Davis, Davis, CA, United States

**Keywords:** obesity, brown adipose tissue, white adipose tissue, oxytocin, food intake

## Abstract

Previous studies have indicated that oxytocin (OT) reduces body weight in diet-induced obese (DIO) rodents through reductions in energy intake and increases in energy expenditure. We recently demonstrated that hindbrain [fourth ventricular (4V)] administration of OT evokes weight loss and elevates interscapular brown adipose tissue temperature (T_*IBAT*_) in DIO rats. What remains unclear is whether OT can be used as an adjunct with other drugs that directly target beta-3 receptors in IBAT to promote BAT thermogenesis and reduce body weight in DIO rats. We hypothesized that the combined treatment of OT and the beta-3 agonist, CL 316243, would produce an additive effect to decrease body weight and adiposity in DIO rats by reducing energy intake and increasing BAT thermogenesis. We assessed the effects of 4V infusions of OT (16 nmol/day) or vehicle (VEH) in combination with daily intraperitoneal injections of CL 316243 (0.5 mg/kg) or VEH on food intake, T_*IBAT*_, body weight and body composition. OT and CL 316243 alone reduced body weight by 7.8 ± 1.3% (*P* < 0.05) and 9.1 ± 2.1% (*P* < 0.05), respectively, but the combined treatment produced more pronounced weight loss (15.5 ± 1.2%; *P* < 0.05) than either treatment alone. These effects were associated with decreased adiposity, adipocyte size, energy intake and increased uncoupling protein 1 (UCP-1) content in epididymal white adipose tissue (EWAT) (*P* < 0.05). In addition, CL 316243 alone (*P* < 0.05) and in combination with OT (*P* < 0.05) elevated T_*IBAT*_ and IBAT UCP-1 content and IBAT thermogenic gene expression. These findings are consistent with the hypothesis that the combined treatment of OT and the beta-3 agonist, CL 316243, produces an additive effect to decrease body weight. The findings from the current study suggest that the effects of the combined treatment on energy intake, fat mass, adipocyte size and browning of EWAT were not additive and appear to be driven, in part, by transient changes in energy intake in response to OT or CL 316243 alone as well as CL 316243-elicited reduction of fat mass and adipocyte size and induction of browning of EWAT.

## Introduction

While it is well appreciated that the hypothalamic nonapeptide, oxytocin (OT), is important in the control of reproductive behavior (Gimpl and Fahrenholz, 2001) and in the formation of prosocial behaviors (i.e., trust, emotion) (Kosfeld et al., 2005; Striepens et al., 2011), less is known about how OT functions in the control of energy balance (Blevins and Baskin, 2015; Lawson, 2017; Lawson et al., 2020; McCormack et al., 2020). While OT elicits weight loss, in part, by reducing food intake, pair-feeding studies suggest that OT’s effects on weight loss cannot be fully explained by its ability to reduce food intake (Deblon et al., 2011; Morton et al., 2012; Altirriba et al., 2015). Recent studies have shown that OT may impact other functions including lipolysis (Deblon et al., 2011; Blevins et al., 2015; Yi et al., 2015) and energy expenditure (Zhang et al., 2011; Zhang and Cai, 2011; Noble et al., 2014; Blevins et al., 2015). OTs effects on lipolysis and energy expenditure in rats may occur directly, in part, through activation of forebrain (Noble et al., 2014; Blevins et al., 2016) or hindbrain (Roberts et al., 2017). OT receptors (OTR) and subsequent increases in sympathetic nervous system outflow to either BAT or white adipose tissue (WAT). More recent findings suggest that OT may also act directly on white adipocytes (Deblon et al., 2011; Yi et al., 2015) where OTR are expressed (Yi et al., 2015). Further studies will need to be done to tease out the significance of discrete OTR populations on these particular functions.

Recent studies suggest that combination therapy (i.e., dual agonists) may be more optimal than monotherapy for sustained weight loss (Frias et al., 2017; Chepurny et al., 2018) (for review see Rodgers et al., 2012). While OT is effective at producing sustained weight loss in DIO rodents (Deblon et al., 2011; Maejima et al., 2011, 2017; Zhang et al., 2011; Zhang and Cai, 2011; Morton et al., 2012; Blevins et al., 2016; Roberts et al., 2017; Edwards et al., 2021), non-human primates (Blevins et al., 2015) and humans (Zhang et al., 2013), its overall effects on weight loss relative to pre-treatment appears to be relatively modest after 4–8 weeks [≈ 4.9% in DIO mice (Roberts et al., 2017), 8.7% in DIO rats (Roberts et al., 2017), 3.3% in DIO rhesus monkeys (Blevins et al., 2015), and 9.3% humans (Zhang et al., 2013)], compared to weight loss achieved in response to long-term (20 weeks to ≥ 1 year) studies in humans treated with cagrilintide (amylin analog) and semaglutide [≈ 17.1% of initial body weight (Enebo et al., 2021)] and FDA-approved drugs such as Qsymia (phentermine + topiramate) [≈ 10.9% of initial body weight; (Allison et al., 2012)]. Despite OT treatment being effective at producing sustained reductions in body weight it has limited efficacy in response to long-term treatment when given as a monotherapy. This may be due, in part, to (1) downregulation of OTRs in specific CNS sites in response to chronic OT treatment (Freeman et al., 2018), or (2) the activation of potent counter-regulatory orexigenic mechanisms in the CNS that drive hunger and energy conservation mechanisms that reduce energy expenditure (EE) to prevent further weight loss and promote weight regain (Myers et al., 2010). Thus, OT in combination with other weight loss agents that act, in part, to reduce food intake and boost energy expenditure may act in an additive fashion to achieve greater weight loss than either treatment given alone.

Similar to OT, the beta-3 receptor agonist, CL 316243, reduces weight gain (Suarez et al., 2014), body weight (Ferrer-Lorente et al., 2010) and adiposity (Suarez et al., 2014; Xiao et al., 2015), in part, through reductions of food intake (Grujic et al., 1997; Suarez et al., 2014) and increases in energy expenditure (Grujic et al., 1997; Suarez et al., 2014; Xiao et al., 2015) in rodents. Furthermore, both OT and CL 316243 increase BAT thermogenesis (Enriori et al., 2011; Mirbolooki et al., 2014; Suarez et al., 2014; Xiao et al., 2015; Roberts et al., 2017; Edwards et al., 2021), a process that results in the stimulation of energy expenditure in the form of heat (Cannon and Nedergaard, 2004) and in some cases, transdifferentiation or *de novo* synthesis of brown adipocytes in white adipose tissue (WAT) (e.g., browning), culminating with increased expression of uncoupling protein-1 (UCP-1) and the production of heat (Cannon and Nedergaard, 2004). In addition to increasing BAT thermogenesis, CL 316243 also increases browning of WAT (Himmshagen et al., 1994; Himms-Hagen et al., 2000; Mottillo et al., 2014; Xiao et al., 2015) in rodent models. Recently, systemic OT treatment has also been shown to increase browning of WAT in mice (Plante et al., 2015; Yuan et al., 2020) although the extent to which OT administration (central or peripheral) promotes browning of WAT in rats and the OTR populations that contribute to these effects in the rat model remains to be determined. Given that the effects of chronic administration of CL 316243 to reduce body weight in C57BL/6J mice appear limited on account of increased food intake (Xiao et al., 2015), OT could therefore be a useful adjunct to offset these effects through its multi-pronged effects to boost BAT thermogenesis and browning of WAT and reduce food intake.

Our initial goal was to test the hypothesis that the combined treatment of OT and the beta-3 agonist, CL 316243, produces an additive effect to decrease body weight and adiposity in DIO rats by reducing energy intake and increasing BAT thermogenesis and browning of EWAT. OT was administered into the fourth ventricle (4V) in order to target hindbrain OTRs based on evidence supporting a role for hindbrain OTRs in the control of food intake (Kirchgessner and Sclafani, 1988; Kirchgessner et al., 1988; Blevins et al., 2003, 2004; Baskin et al., 2010; Ho et al., 2014; Ong et al., 2015, 2017; Roberts et al., 2017; Edwards et al., 2021), thermogenesis (Ong et al., 2017; Roberts et al., 2017; Edwards et al., 2021) and in OT-elicited weight loss (Roberts et al., 2017; Edwards et al., 2021). We subsequently examined the effects of chronic minipump infusions of OT (16 nmol/day) or vehicle (VEH) into the 4V in combination with daily IP injections of lower escalating doses of CL 316243 (0.001–0.5 mg/kg) on energy intake, IBAT temperature (T_*IBAT*_; as a functional surrogate of BAT thermogenesis), body weight and body composition. We also examined if OT (16 nmol/day) or vehicle in combination with a longer duration (≈ 3-week/single dose) treatment of a single dose (0.5 mg/kg) of CL 316243 was an effective strategy to reduce body weight and adiposity and if these effects were associated with reductions of adipocyte size and energy intake as well as increased BAT thermogenesis and browning of epididymal white adipose tissue (EWAT).

## Materials and Methods

### Animals

Adult male Long-Evans rats [≈ 8–9 weeks old, 292–349 grams at start of high fat dietary (HFD) intervention/≈ 8–10 months old, 526–929 g body weight at study onset] were initially obtained from Envigo (Indianapolis, IN, United States) and maintained for at least 4 months on a high fat diet (HFD) prior to study onset. All animals were housed individually in Plexiglas cages in a temperature-controlled room (22 ± 2°C) under a 12:12-h light-dark cycle. All rats were maintained on a 1 a.m./1 p.m. light cycle. Rats had *ad libitum* access to water and a HFD providing 60% kcal from fat (approximately 6.8% kcal from sucrose and 8.9% of the diet from sucrose) (Research Diets, D12492, New Brunswick, NJ, United States). The research protocols were approved both by the Institutional Animal Care and Use Committee of the Veterans Affairs Puget Sound Health Care System (VAPSHCS) and the University of Washington in accordance with NIH Guidelines for the Care and Use of Animals.

### Drug Preparation

Fresh solutions of OT acetate salt (Bachem Americas, Inc., Torrance, CA, United States) were solubilized in sterile water and subsequently primed in sterile 0.9% saline at 37°C for approximately 40 h prior to minipump implantation based on manufacturer’s recommended priming instructions for ALZET^®^ model 2004 minipumps. CL 316243 (Tocris/Bio-Techne Corporation, Minneapolis, MN, United States) was solubilized in sterile water each day of each experiment.

### 4V Cannulations for Chronic Infusions

Animals were implanted with a cannula within the 4V with a side port that was connected to an osmotic minipump (model 2004, DURECT Corporation) as previously described (Blevins et al., 2015; Roberts et al., 2017). While under isoflurane anesthesia rats were placed in a stereotaxic apparatus with the incisor bar positioned 3.3 mm below the interaural line. A guide cannula (30-gauge; P1 Technologies) was stereotaxically directed toward the 4V [7.3 mm caudal to bregma; 0 mm lateral to the midline, and 8.6 mm ventral to the skull surface] and fastened to the skull using stainless steel screws and dental acrylic. Plastic Tygon^TM^ Microbore Tubing (2.4″; 0.020″ × 0.060″OD; Cole-Parmer) was tunneled subcutaneously along the middle of the back before being attached to the sidearm (21 gauge) osmotic minipump-cannula assembly. In addition, a pin plug (22-gauge stainless steel; Instech Laboratories, Inc.) was temporarily placed into the distal end of the plastic tubing during the post-operative recovery period prior to being replaced by an osmotic minipump (DURECT Corporation) containing saline or OT. Animals were treated with the analgesic ketoprofen (2 mg/kg; Fort Dodge Animal Health) and the antibiotic enrofloxacin (5 mg/kg; Bayer Healthcare LLC., Animal Health Division Shawnee Mission, KS, United States) at the completion of the 4V cannulations and were allowed to recover at least 10 days prior to being implanted with osmotic minipumps.

### Implantation of Temperature Transponders Underneath IBAT

Rats were anesthetized with isoflurane prior to having the dorsal surface along the upper midline of the back shaved and scrubbed with 70% ethanol followed by betadine swabs as previously described (Roberts et al., 2017). Following an incision (1″) along the midline of the interscapular area a temperature transponder (14 mm long/2 mm wide) (HTEC IPTT-300; BIO MEDIC DATA SYSTEMS, INC., Seaford, DE, United States) was implanted underneath the left IBAT pad as previously described (Brito et al., 2007; Vaughan et al., 2011; Roberts et al., 2017). The transponder was subsequently secured in place by suturing it to the brown fat pad with sterile silk suture. The interscapular incision was closed with Nylon sutures (5-0), which were removed in awake animals approximately 10–14 days post-surgery. HTEC IPTT-300 transponders were used in place of IPTT-300 transponders to enhance accuracy in our measurements as previously described (Roberts et al., 2017).

### Acute IP Injections and Measurements of T_*IBAT*_

CL 316243 (or saline vehicle; 0.1 ml/kg injection volume) was administered immediately prior to the start of the dark cycle following 4 h of food deprivation. Animals remained without access to food for an additional 1 (Study 2–3) or 4 h (Study 1) during the course of the T_*IBAT*_ measurements. A handheld reader (DAS-8007-IUS Reader System; BIO MEDIC DATA SYSTEMS, INC.) was used to collect measurements of T_*IBAT*_. For dose-response studies (Study 1), rats underwent all treatments in a randomized order separated by at least 7–8 days between treatments. Dose-response studies were conducted in order to determine a minimal dose of the beta-3 receptor agonist, CL 316243, to use in the chronic administration studies (Studies 2–4).

### Body Composition

Determinations of lean body mass and fat mass were made on un-anesthetized rats by quantitative magnetic resonance using an EchoMRI 4-in-1–700^TM^ instrument (Echo Medical Systems, Houston, TX, United States) at the VAPSHCS Rodent Metabolic Phenotyping Core. Measurements were taken prior to 4V cannulations and minipump implantations as well as at the end of the infusion period.

## Study Protocols

### Study 1: Dose-Response Effects of Acute Beta-3 Receptor Agonist CL 316243 (0.001–1 mg/kg) on Body Weight, Energy Intake and T_*IBAT*_ in Male DIO Rats

Rats (≈ 8 mo old; 599–901 g at start of study) were fed *ad libitum* and maintained on HFD for approximately 5.5 months prior to receiving transponder implantations. Animals were allowed to recover for at least 1 week during which time they were adapted to daily handling and mock injections. On an experimental day, animals received CL 316243 (0.001, 0.01, 0.1, or 1 mg/kg, IP) or vehicle (sterile water, IP) (1×/experimental day at 8-day intervals) immediately prior to the start of the dark period in a crossover design such that each animal served as its own control and received each treatment. These doses were previously found to reduce weight gain, adiposity, food intake and elevate T_*IBAT*_ in lean rats following systemic (IP) administration (Suarez et al., 2014). T_*IBAT*_ was measured at baseline (−4 h; 9:00 a.m.), immediately prior to IP injections (0 h; 12:45–1:00 p.m.), and at 0.25, 0.5, 0.75, 1, 1.25, 1.5, 2, 3, 4, and 20-h post-injection (9:00 a.m.). This dose range was based on doses of CL 316243 found to be effective at reducing food intake and weight gain in rats (Suarez et al., 2014) (paradigm shown in Supplementary Figure 1).

### Study 2: Effect of Chronic 4V OT Infusions (16 nmol/day) and Systemic Beta-3 Receptor Agonist (CL 316243) Administration (0.001–0.5 mg/kg) on Daily Body Weight, Body Adiposity, Energy Intake and T_*IBAT*_ in Male DIO Rats

Rats (≈ 9 mo old; 595–870 g at start of study) were fed *ad libitum* and maintained on HFD for approximately 5.5 months prior to receiving implantations of temperature transponders underneath the left IBAT pad in addition to 4V cannulas and 28-day minipumps to infuse vehicle or OT (16 nmol/day) over 28 days, respectively. We have previously found that 4V infusions of OT at this dose evoked weight loss in DIO rats (Roberts et al., 2017). After having matched animals for OT-elicited reductions in body weight (infusion day 4), DIO rats subsequently received 1× daily IP injections of VEH or CL 316243 as part of a dose escalation study (0.001, 0.01, 0.05, and 0.5 mg/kg). T_*IBAT*_ was measured daily at baseline (−4 h; 9:00 a.m.), immediately prior to IP injections (0 h; 12:45–1:00 p.m.), and at 0.25, 0.5, 0.75, 1, 20, and 24-h post-injection. In addition, daily food intake and body weight were also tracked for 28 days. Data from animals that received different doses of CL-31643 were pooled together and analyzed over the 28-day infusion period (paradigm shown in Supplementary Figure 1).

### Study 3: Effect of Chronic 4V OT Infusions (16 nmol/day) and Systemic Beta-3 Receptor Agonist (CL 31643) Administration (0.5 mg/kg) on Body Weight, Body Adiposity, Energy Intake and T_*IBAT*_ in Male DIO Rats

Rats (≈ 10 mo old; 526–929 g at start of study) were fed *ad libitum* and maintained on HFD for approximately 7.5 months prior to receiving implantations of temperature transponders underneath the left IBAT pad in addition to 4V cannulas and 28-day minipumps to infuse vehicle or OT (16 nmol/day) over 28 days, respectively. After having matched animals for OT-elicited reductions in body weight (infusion day 7), DIO rats subsequently received 1× daily IP injections of VEH or CL 316243 (0.5 mg/kg). We selected this dose because it elevated T_*IBAT*_ at doses that failed to produce elevations in heart rate in lean rats (Malinowska and Schlicker, 1997). T_*IBAT*_ was measured daily at baseline (−4 h; 9:00 a.m.), immediately prior to IP injections (0 h; 12:45–1:00 p.m.), and at 0.25, 0.5, 0.75, 1, 20, and 24-h post-injection. In addition, daily food intake and body weight were also tracked for 28 days. Data from animals that received the single dose of CL-31643 were analyzed over the 28-day infusion period (paradigm shown in Supplementary Figure 1).

### Adipose Tissue Processing for Adipocyte Size and UCP-1 Analysis

IBAT, IWAT and EWAT depots were collected at the end of the infusion period in rats from Study 3. Rats from each group were euthanized following a 3-h fast. Rats were euthanized with intraperitoneal injections of ketamine cocktail [ketamine hydrochloride (214.3 mg/kg), xylazine (10.71 mg/kg) and acepromazine (3.3 mg/kg) in an injection volume up to 1 mL/rat] and transcardially exsanguinated with PBS followed by perfusion with 4% paraformaldehyde in 0.1 M PBS. Adipose tissue (IBAT, IWAT, and EWAT) was dissected and placed in 4% paraformaldehyde-PBS for 24 h and then placed in 70% ethanol (EtOH) prior to paraffin embedding. Sections (5 μm) sampled were obtained using a rotary microtome, slide-mounted using a floatation water bath (37°C), and baked for 30 min at 60°C to give approximately 15–16 slides/fat depot with two sections/slide.

### Adipocyte Size Analysis and UCP-1 Staining

Adipocyte size analysis was performed on deparaffinized and digitized IWAT and EWAT sections. The average cell area from two randomized photomicrographs was determined using the built-in particle counting method of ImageJ software (National Institutes of Health, Bethesda, MD, United States). Fixed (4% PFA), paraffin-embedded adipose tissue was sectioned and stained with a primary rabbit anti-UCP-1 antibody [1:100; Abcam, Cambridge, MA, United States (#ab10983/RRID:AB_2241462] as has been previously described in lean C57BL/6J mice (Cao et al., 2019) and both lean and DIO C57BL/6 mice after having been screened in both IBAT and IWAT of Ucp1^+/–^ and Ucp1^–/–^ mice (Veniant et al., 2015). Immunostaining specificity controls included omission of the primary antibody and replacement of the primary antibody with normal rabbit serum at the same dilution as the respective primary antibody (Supplementary Figure 2). Area quantification for UCP1 staining was performed on digital images of immunostained tissue sections using image analysis software (Image Pro Plus software, Media Cybernetics, Rockville, MD, United States). Slides were visualized using bright field on an Olympus BX51 microscope (Olympus Corporation of the Americas; Center Valley, PA, United States) and photographed using a Canon EOS 5D SR DSLR (Canon U.S.A., Inc., Melville, NY, United States) camera at 100× magnification. Values for each tissue within a treatment were averaged to obtain the mean of the treatment group.

### Blood Collection

Blood was collected from 6-h fasted rats at 2-h post-CL 316243 [0. 5 mg/kg (Study 2 and Study 3) or 1 mg/kg (Study 1)] or VEH administration within a 2-h window toward the end of the light cycle (10:00 a.m.–12:00 p.m.) as previously described in DIO CD^®^ IGS rats and mice (Blevins et al., 2016; Roberts et al., 2017). Treatment groups were counterbalanced at time of euthanasia to avoid time of day bias. Blood samples [up to 3 mL] were collected immediately prior to transcardial perfusion by cardiac puncture in chilled K2 EDTA Microtainer Tubes (Becton-Dickinson, Franklin Lakes, NJ, United States). Whole blood was centrifuged at 6,000 rpm for 1.5-min at 4°C; plasma was removed, aliquoted and stored at −80°C for subsequent analysis.

### Plasma Hormone Measurements

Plasma leptin and insulin were measured using electrochemiluminescence detection [Meso Scale Discovery (MSD^®^), Rockville, MD, United States] using established procedures (Bremer et al., 2011; Roberts et al., 2017). Intra-assay coefficient of variation (CV) for leptin was 2.7 and 3.2% for insulin. The range of detectability for the leptin assay is 0.137–100 ng/mL and 0.069–50 ng/mL for insulin. Plasma fibroblast growth factor-21 (FGF-21) (R&D Systems, Minneapolis, MN, United States) and irisin (AdipoGen, San Diego, CA, United States) levels were determined by ELISA. The intra-assay CV for FGF-21 and irisin were 4.5 and 8.4%, respectively; the ranges of detectability were 31.3–2000 pg/mL (FGF-21) and 0.078–5 μg/mL (irisin). Plasma adiponectin was also measured using electrochemiluminescence detection [Meso Scale Discovery (MSD^®^), Rockville, MD, United States] using established procedures (Bremer et al., 2011; Roberts et al., 2017). Intra-assay CV for adiponectin was 1.1%. The range of detectability for the adiponectin assay is 2.8–178 ng/mL. The data were normalized to historical values using a pooled plasma quality control sample that was assayed in each plate.

### Blood Glucose and Lipid Measurements

Blood was collected for glucose measurements by tail vein nick at 2-h post-CL 316243 or VEH administration and measured with a glucometer using the AlphaTRAK 2 blood glucose monitoring system (Abbott Laboratories, Abbott Park, IL, United States) (Blevins et al., 2012). Total cholesterol (TC) [Fisher Diagnostics (Middletown, VA, United States)] and free fatty acids (FFAs) (Wako Chemicals USA, Inc., Richmond, VA, United States) were measured using an enzymatic based kits. Intra-assay CVs for TC and FFAs were 1.4 and 2.3%, respectively. These assay procedures have been validated for rodents (Cummings et al., 2008).

### Tissue Collection for Quantitative Real-time PCR (qPCR)

IBAT and IWAT tissue was collected from 6-h fasted rats at 2-h post-CL 316243 (1 mg/kg) or VEH administration for Study 1. Tissue was collected in an identical manner for animals in Study 2 with the exception that animals received a lower dose of CL 316243 (0.5 mg/kg) or VEH in combination with chronic 4V infusions of OT (16 nmol/day) or VEH. IBAT and IWAT were collected within a 2-h window toward the end of the light cycle (10:00 a.m.–12:00 p.m.) as previously described in DIO CD^®^ IGS/Long-Evans rats and C57BL/6J mice (Blevins et al., 2016; Roberts et al., 2017; Edwards et al., 2021). Tissue was rapidly removed, wrapped in foil and frozen in liquid N2. Samples were stored frozen at −80°C until analysis.

### qPCR

RNA extracted from samples of IBAT and IWAT (Studies 1–2) were analyzed using the RNeasy Lipid Mini Kit (Qiagen Sciences Inc., Germantown, MD, United States) followed by reverse transcription into cDNA using a high-capacity cDNA archive kit (Applied Biosystems, Foster City, CA, United States). Quantitative analysis for relative levels of mRNA in the RNA extracts was measured in duplicate by qPCR on an Applied Biosystems 7500 Real-Time PCR system (Thermo Fisher Scientific, Waltham, MA, United States) and normalized to the cycle threshold value of Nono mRNA in each sample. The TaqMan^®^ probes used in the study were Thermo Fisher Scientific Gene Expression Assay probes. The probe for rat Nono (Rn01418995_g1), UCP-1 (catalog no. Rn00562126_m1), UCP-2 (catalog no. Rn01754856_m1), UCP-3 (catalog no. Rn00565874_m1), beta-1 adrenergic receptor (Adrb1; catalog no. Rn00824536_s1), beta-2 adrenergic receptor (Adrb2; catalog no. Rn00560650_s1), beta-3 adrenergic receptor (Adrb3; catalog no. Rn01478698_g1), alpha-2 adrenergic receptor (Adra2a; catalog no. Rn00562488_s1),type 2 deiodinase (D2) (Dio2; catalog no. Rn00581867_m1), cytochrome c oxidase subunit 8b (Cox8b; catalog no. Rn00562884_m1), G-protein coupled receptor 120 (Gpr120; catalog no. Rn01759772_m1), bone morphogenetic protein 8b (bmp8b; catalog no. Rn01516089_gH), cell death-inducing DNA fragmentation factor alpha-like effector A (Cidea; catalog no. Rn04181355_m1), peroxisome proliferator-activated receptor gamma coactivator 1 alpha (Ppargc1a; catalog no. Rn00580241_m1) were acquired from Thermo Fisher Scientific. Relative amounts of target mRNA were determined using the Comparative C_*T*_ or 2-^ΔΔ*C*^_*T*_ method (Livak and Schmittgen, 2001) following adjustment for the housekeeping gene.

### Transponder Placement

All temperature transponders were confirmed to have remained underneath the IBAT depot at the conclusion of the study.

### Statistical Analyses

All results are expressed as means ± SE. Comparisons between multiple groups involving between subjects designs were made using one- or two-way ANOVA as appropriate, followed by a *post hoc* Fisher’s least significant difference test. Comparisons involving within-subjects designs were made using a one-way repeated-measures ANOVA followed by a *post hoc* Bonferroni Test. Analyses were performed using the statistical program SYSTAT (SYSTAT Software, Point Richmond, CA, United States). Differences were considered significant at *P* < 0.05, 2-tailed.

## Results

### Study 1: Dose-Response Effects of Acute Beta-3 Receptor Agonist CL 316243 (0.001–1 mg/kg) on Body Weight, Energy Intake and T_*IBAT*_ in Male DIO Rats

The goal of this study was to identify a dose range of the beta-3 receptor agonist, CL 316243, that resulted in weight loss, reduced energy intake and an elevation in IBAT temperature in DIO rats. The effective dosing data from this study was used to select a dose range (0.001–1 mg/kg, IP) for use in the subsequent chronic dose escalation study (Study 2). By design, DIO rats were obese as determined by both body weight (736 ± 29 g) and adiposity (267 ± 22 g fat mass; 36.1 ± 1.6% adiposity) after maintenance on the HFD for approximately 5.5 months.

#### IBAT Temperature

There was a significant main effect of CL 316243 to elevate T_*IBAT*_ at 0.25- [*F*(4,35) = 13.482, *P* < 0.05], 0.5- [*F*(4,35) = 17.150, *P* < 0.05], 0.75- [*F*(4,36) = 21.950, *P* < 0.05], 1- [*F*(4,36) = 14.213, *P* < 0.05], 1.25- [*F*(4,36) = 154.298, *P* < 0.05], 1.5- [*F*(4,35) = 19.618, *P* < 0.05], 1.75- [*F*(4,36) = 16.762, *P* < 0.05], 2- [*F*(4,36) = 18.267, *P* < 0.05], 3-[*F*(4,36) = 15.131, *P* < 0.05], 4- [*F*(4,36) = 17.853, *P* < 0.05] and 20-h post-injection [*F*(4,36) = 35.435, *P* < 0.05]. We also found a significant effect of time [*F*(11,462) = 86.673, *P* < 0.05] and a significant interactive effect between time and dose [*F*(44,462) = 4.573, *P* < 0.05] across 12 time points over the 20-h measurement period.

**FIGURE 1:**
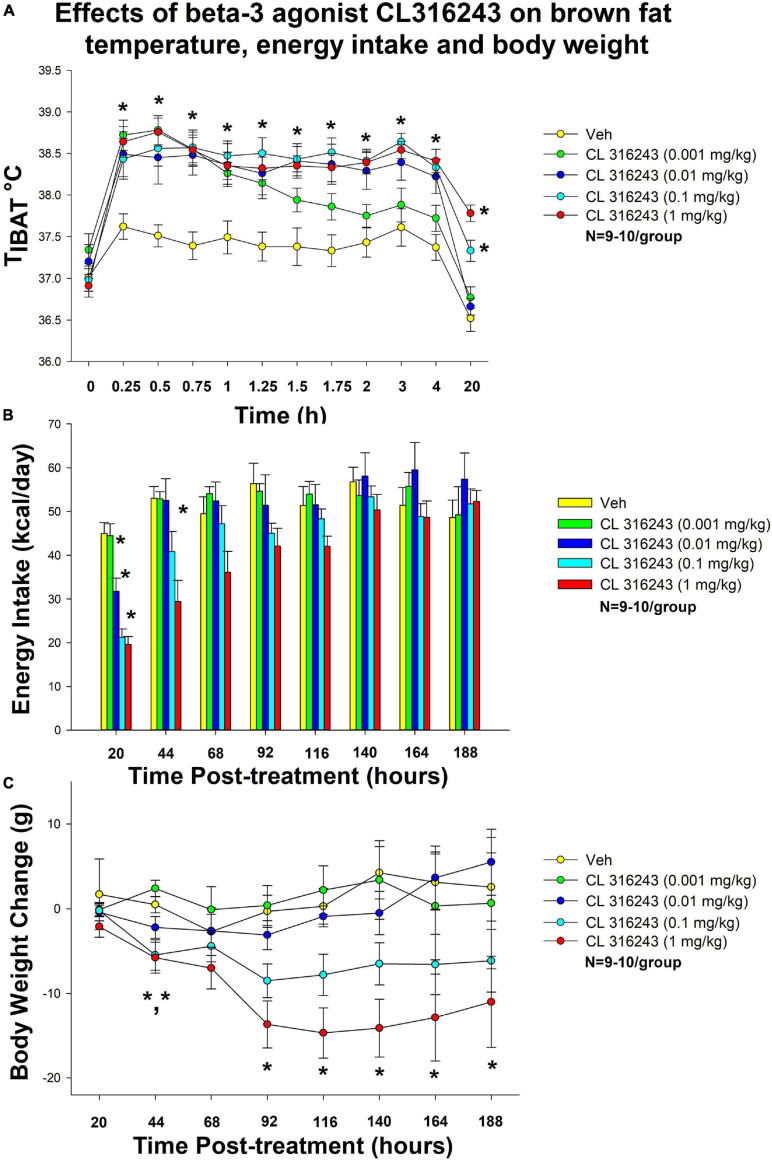
Dose-response effects of the beta-3 receptor agonist, CL 316243, on body weight, food intake, and T_*IBAT*_ in male DIO rats. *Ad libitum* fed rats were either maintained on HFD (60% kcal from fat; *N* = 9–10/group) for approximately 5.5 months prior to receiving IP injections of CL 316243 (0.001–1 mg/kg) or vehicle (sterile water) where each animal received each treatment 1× every 8 days. **(A)** Effect of CL 316243 on T_IBAT_ in DIO rats. **(B)** Effect of CL 316243 on energy intake (kcal/day) in DIO rats. **(C)** Effect of CL 316243 on change in body weight in HFD-fed DIO rats. Data are expressed as mean_SEM. *P < 0.05, **(A)** CL 316243 (0.01–1 mg/kg) vs. vehicle on T_IBAT_ at 0.25, 0.5, 0.75, 1, 1.25, 1.5, 1.75, 2, 3, 4, and 20-h post-injection and CL 316243 (0.001 mg/kg) vs. vehicle on T_IBAT_ at 0.25, 0.5, 0.75, 1, 1.25, and 1.5-h post-injection. CL 316243 (0.1 and 1 mg/kg) vs. vehicle on T_IBAT_ at 20-h post-injection. *P < 0.05, **(C)** CL 316243 (0.1 and 1 mg/kg) vs. vehicle on body weight change at 44-h post-injection and CL 316243 (1 mg/kg) vs. vehicle on body weight change at 92-, 116-, 140-, 164-, and 188-h post-injection.

Specifically, systemic injections of CL 316243 treatment increased in T_*IBAT*_ (Figure 1A). CL 316243 (0.01–1 mg/kg) increased T_*IBAT*_ at 15, 30, 45, 60, 75, 90, 105, 120, 180, and 240-min post-injection. The lowest dose (0.001 mg/kg) also stimulated T_*IBAT*_ at 15, 30, 45, 60, 75, and 90-min post-injection. CL 316243 also stimulated T_*IBAT*_ at 20-h post-injection at 0.1 and 1 mg/kg (*P* < 0.05).

### Energy Intake

There was a significant main effect of CL 316243 to reduce 20-h food intake at 1 [*F*(4,36) = 22.318, *P* < 0.05], 2 [*F*(4,34) = 5.271, *P* < 0.05] and 3 days post-injection [*F*(4,35) = 3.788, *P* < 0.05]. Specifically, CL 316243 reduced 20-h energy intake at 0.01, 0.1, and 1 mg/kg (*P* < 0.05) by 29, 53, and 56% but the lowest dose (0.001 mg/kg) failed to significantly reduce 20-h energy intake (*P* = NS) (Figure 1B). The high dose also reduced energy intake by 45% (1 mg/kg, *P* < 0.05) at 44-h post-injection.

### Body Weight

There was no overall significant main effect of CL 316243 to reduce body weight gain at any of the doses examined at 20-h post-injection [*F*(4,36) = 0.420, *P* = NS] but there was a main effect of CL 316243 to reduce weight gain at 44- [*F*(4,35) = 7.190, *P* < 0.05], 92- [*F*(4,35) = 6.907, *P* < 0.05], 116- [*F*(4,35) = 7.718, *P* < 0.05], 140- [*F*(4,35) = 7.446, *P* < 0.05], 164- [*F*(4,35) = 7.194, *P* < 0.05], and 188-h post-injection [*F*(4,35) = 5.782, *P* < 0.05]. Specifically, both high doses (0.1 and 1 mg/kg) reduced the body weight gain at 44-h (*P* < 0.05) while the highest dose (1 mg/kg) reduced body weight gain at 92-, 116-, 140-, 164-, and 188-h post-injection (*P* < 0.05 vs. VEH) (Figure 1C).

### Gene Expression Data

#### IBAT

As an additional readout of CL 316243-elicited thermogenic effects in IBAT, relative levels of mRNA for UCP-1 and Ppargc1a were compared by PCR in response to CL 316243 (1 mg/kg) or VEH treatment at 2-h post-injection (Table 1A). Consistent with published findings in mice and rats, CL 316243 injections increased T_*IBAT*_ between 0.25 and 2-h post-injection (Table 1C; *P* < 0.05) and elevated relative levels of the thermogenic markers UCP-1 (*P* < 0.05) (Rosell et al., 2014; Suarez et al., 2014; Gonzalez-Hurtado et al., 2018), DIO2 (*P* < 0.05) (de Jong et al., 2017) and Ppargc1a mRNA (Rosell et al., 2014) (*P* < 0.05) at 2-h post-injection. CL 316243 also caused a downregulation of the beta-3 receptor @ 2-h post-injection (*P* < 0.05). This finding is consistent with other reports that have found norepinephrine and cold exposure to reduce beta-3 receptor mRNA expression in mouse brown adipocytes (Bengtsson et al., 2000) and mouse IBAT (Kasahara et al., 2013, 2015). In addition, we found that CL 316243 tended to increase IBAT beta-1 receptor expression (*P* = 0.071). This finding is also consistent with earlier work that reported norepinephrine and CL 316243 to increase IBAT beta-1 receptor mRNA expression in mouse brown adipocytes (Bengtsson et al., 2000) and wild-type mice (de Jong et al., 2017), respectively.

**TABLE 1:** Changes in IBAT and IWAT gene expression following CL 316243 treatment in male DIO rats.

(A) Changes in IBAT gene expression following CL 316243 (1 mg/kg) treatment in male DIO rats						
Treatment		VEH		CL 316243		
**IBAT**						
*Adra2a*		1.0 ± 0.3^*a*^		1.0 ± 0.2^*a*^		
*beta1ar*		1.0 ± 0.1^*a*^		2.3 ± 0.6^*a*^		
*beta2ar*		1.0 ± 0.2^*a*^		1.1 ± 0.3^*a*^		
*beta3ar*		1.0 ± 0.2^*a*^		0.5 ± 0.1^*b*^		
*UCP1*		1.0 ± 0.2^*a*^		2.0 ± 0.1^*b*^		
*UCP2*		1.0 ± 0.2^*a*^		0.9 ± 0.1^*a*^		
*UCP3*		1.0 ± 0.2^*a*^		0.9 ± 0.2^*a*^		
*bmp8b*		1.0 ± 0.2^*a*^		0.8 ± 0.2^*a*^		
*Cidea*		1.0 ± 0.1^*a*^		1.1 ± 0.2^*a*^		
*Gpr120*		1.0 ± 0.2^*a*^		2.6 ± 0.1^*a*^		
*Cox8b*		1.0 ± 0.1^*a*^		1.4 ± 0.2^*a*^		
*DIO2*		1.0 ± 0.3^*a*^		12.4 ± 1.9^*b*^		
*Ppargc1a*		1.0 ± 0.2^*a*^		10.9 ± 2.0^*b*^		
**(B) Changes in IWAT gene expression following CL 316243 (1 mg/kg) treatment in male DIO rats**						
**Treatment**		**VEH**		**CL 316243**		

**IWAT**						
*Adra2a*		1.0 ± 1.0^*a*^		0.03 ± 0.02^*a*^		
*beta1ar*		1.0 ± 0.5^*a*^		0.8 ± 0.2^*a*^		
*beta2ar*		1.0 ± 0.2^*a*^		0.9 ± 0.1^*a*^		
*beta3ar*		1.0 ± 0.7^*a*^		0.6 ± 0.1^*a*^		
*UCP1*		1.0 ± 0.3^*a*^		1.3 ± 0.6^*a*^		
*UCP2*		1.0 ± 0.3^*a*^		0.8 ± 0.1^*a*^		
*UCP3*		1.0 ± 0.5^*a*^		2.2 ± 0.4^*a*^		
*bmp8b*		1.0 ± 0.3^*a*^		0.8 ± 0.2^*a*^		
*Cidea*		1.0 ± 0.3^*a*^		2.2 ± 0.3^*b*^		
*Gpr120*		1.0 ± 0.3^*a*^		3.3 ± 0.9^*a*^		
*Cox8b*		1.0 ± 0.1^*a*^		11.7 ± 6.3^*a*^		
*DIO2*		1.0 ± 0.7^*a*^		2.4 ± 1.0^*a*^		
*Ppargc1a*		1.0 ± 0.3^*a*^		1.9 ± 0.7^*a*^		
**(C) Changes in T_*IBAT*_ Following CL 316243 treatment in male DIO rats**						
**IP**	**0 min**	**15 min**	**30 min**	**45 min**	**60 min**	**120 min**

	**Temp (C°)**	**Temp (C°)**	**Temp (C°)**	**Temp (C°)**	**Temp (C°)**	**Temp (C°)**

**VEH**	37.1 ± 0.1	37.4 ± 0.1	37.3 ± 0.1	37.3 ± 0.2	37.1 ± 0.1	37.5 ± 0.2
**CL 316243**	37.0 ± 0.1	38.7 ± 0.3*	38.4 ± 0.1*	38.1 ± 0.2*	38.1 ± 0.3*	38.4 ± 0.2*

*(A) IBAT, (B) IWAT and (C) T_IBAT_. Data are expressed as mean ± SEM. Different letters denote significant differences between treatments (*P* < 0.05).*

Data are expressed as mean ± SEM. *P < 0.05 vs. VEH.

*Shared letters are not significantly different from one another *N* = 4–5/group.*

#### IWAT

Consistent with previous findings in mice, we found that that CL 316243 (1 mg/kg) also tended to elevate Gpr120 (Rosell et al., 2014) (0.05 < *P* < 0.01) and UCP-3 (Yoshitomi et al., 1998) (0.05 < *P* < 0.01) and produced a significant elevation of Cidea (Gonzalez-Hurtado et al., 2018; Fujimoto et al., 2019; Table 1B; *P* < 0.05). Collectively, these findings are consistent with the ability of CL 316243 to stimulate thermogenesis and elicit “browning” of WAT.

### Study 2: Effect of Chronic 4V OT Infusions (16 nmol/day) and Systemic Beta-3 Receptor Agonist (CL 316243) Administration (0.001–0.5 mg/kg) on Daily Body Weight, Body Adiposity and Energy Intake in Male DIO Rats

The goal of this study was to identify a dose of CL 316243 to use in Study 3A that could be used in combination with OT to result in an additive reduction of body weight and adiposity in DIO rats. The data from this study were used to select a dose (0.5 mg/kg, IP) for use in the subsequent chronic treatment (single dose) study (Study 3). By design, DIO rats were obese as determined by both body weight (751 ± 37 g) and adiposity (284 ± 16 g fat mass; 37.4 ± 1.2% adiposity) after maintenance on the HFD for approximately 5.5 months. Prior to the onset of CL 316243 treatment on infusion day 4, both OT treatment groups were matched for OT-elicited reductions of weight gain.

**FIGURE 2:**
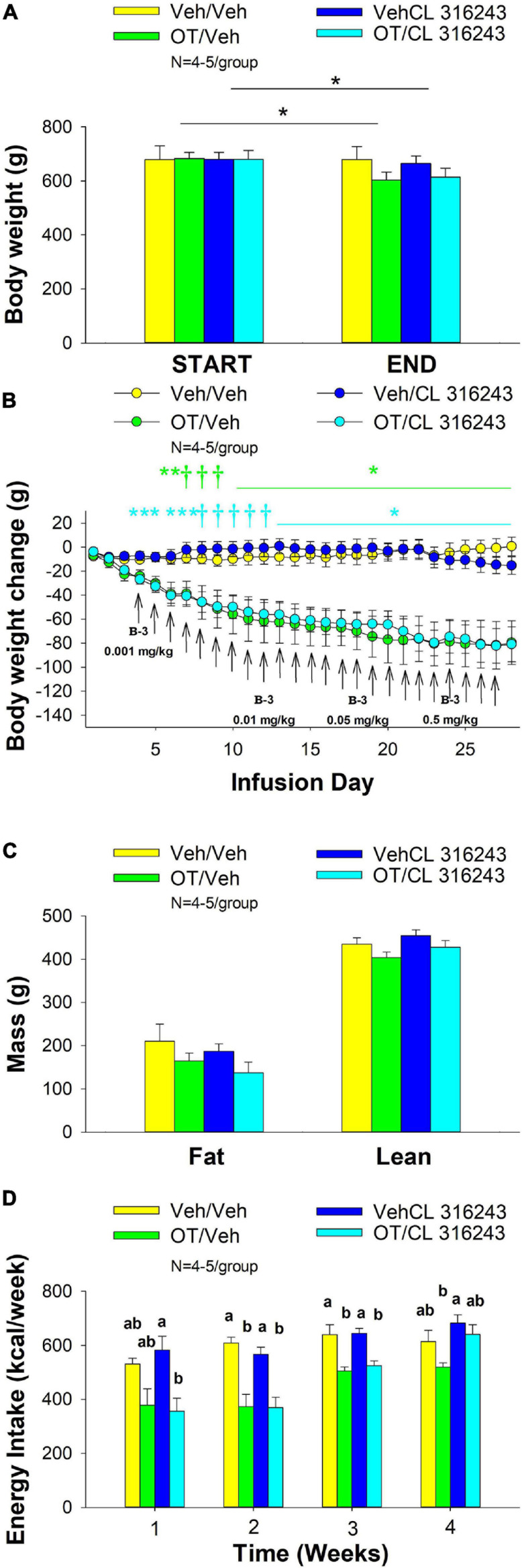
**(A–D)** Effect of chronic 4V OT infusions (16 nmol/day) and systemic beta-3 receptor agonist (CL 316243) administration (0.001–0.5 mg/kg) on daily body weight, body adiposity and energy intake in male DIO rats. *Ad libitum* fed rats were maintained on HFD (60% kcal from fat; *N* = 4–5/group) for approximately 6.5 months prior to receiving continuous infusions of vehicle or OT (16 nmol/day) in combination with increasing doses of CL 316243 (0.001–0.5 mg/kg). **(A)** Change in body weight in HFD-fed DIO rats; **(B)** Change in body weight gain in HFD-fed DIO rats; **(C)** Change in fat mass and lean mass in HFD-fed DIO rats; **(D)** Weekly energy intake (kcal/week) in HFD-fed DIO rats. ↑ indicate 1× daily injections. Different letters denote significant differences between treatments. **(B)** Colored bars represent specific group comparisons vs. vehicle. Data are expressed as mean ± SEM. *P < 0.05, **P = 0.05, ^†^0.05 < *P* < 0.1 OT vs. vehicle or baseline (pre-treatment; **A**).

4V OT alone or the combination of OT and CL 316243 treatment reduced body weight by ≈ 11.7 and 9.7% relative to OT and CL 316243 pre-treatment, respectively (*P* < 0.05) (Figure 2A) and weight gain (Figure 2B) throughout the 28-day infusion period. The combined treatment (OT and CL 316243) reduced weight gain on days 5–6 and 13–28 (*P* < 0.05). Weight gain was also reduced in the OT alone treatment group between days 5 and 10–28 (*P* < 0.05) but CL 316243 alone failed to impact body weight at any point during the study (*P* = NS). While there was a significant difference between CL 316243 alone and the combined treatment (OT and CL 316243) on body weight there was no difference in the effectiveness of OT alone and the combined treatment to reduce weight gain (*P* = NS) at any point during the study. There was also no significant effect of the treatments on fat mass or lean body mass (Figure 2C; *P* = NS).

Oxytocin alone and in combination with CL 316243 reduced energy intake during weeks 2 and 3 but CL 316243 alone failed to impact energy intake at any time point at the specific dosed in Study 2. There were also no differences between OT in combination with CL 316243 and OT alone on energy intake over weeks 2–3. In contrast, there were differences between OT in combination with CL 316243 and CL 316243 alone as well as between OT alone and CL 316243 alone (Figure 2D; *P* < 0.05). Collectively, these findings suggest that OT is driving the reduction in energy intake when combined with doses of CL 316243 used in Study 2.

Two-way ANOVA also failed to reveal a significant interactive effect between OT and CL 316243 on final body weight, reduction of weight gain or weekly energy intake. These findings suggest that OT does not interact with CL 316243 to impact body weight or energy intake at the doses used in the current study (Seeley and Moran, 2002).

As a positive control to confirm effectiveness of CL-316243 over time, we measured the T_*IBAT*_ response to CL-316243 alone or in combination with OT throughout the course of the IP injection study. We initially confirmed that there was no difference in T_*IBAT*_ at baseline (0; immediately prior to injections) on days 1 or 24 (Tables 2A,B). Repeated measures ANOVA (IP injection days 1, 24) indicated there was a significant main effect of CL 316243 alone to elevate T_*IBAT*_ at 0.25- [*F*(1,7) = 13.299, *P* < 0.01], 0.5- [*F*(1,7) = 18.399, *P* < 0.05], 0.75- [*F*(1,7) = 23.154, *P* < 0.05], and 1-h post-injection [*F*(1,7) = 14.077, *P* < 0.05]. CL 316243 increased T_*IBAT*_ at 0. 25-, 0. 5-, 0. 75-, and 1-h post-injection relative to vehicle treatment at 0. 25-, 0. 5-, 0. 75-, and 1-h (*P* < 0.05) on day 1. Similarly, CL 316243 also increased T_*IBAT*_ at 0. 25-, 0. 5-, 0.75- and 1-h post-injection relative to vehicle treatment at 0. 25-, 0. 5-, 0. 75-, and 1-h (*P* < 0.05) on day 24. In addition, repeated measures ANOVA (IP injection days 1, 24) indicated a near significant or significant main effect of OT to elevate T_*IBAT*_ at 0.25- [*F*(1,8) = 3.447, *P* = 0.085], 0.75- [*F*(1,8) = 10.830, *P* < 0.05], and 1-h [*F*(1,8) = 8.824, *P* < 0.05] (Tables 2A,B). OT tended to increase T_*IBAT*_ at 0.25- (*P* = 0.054), 0.75- (*P* < 0.05), 1- (*P* < 0.05), and 24-h post-injection (*P* < 0.05) relative to 4V vehicle treatment on day 1. OT also increased T_*IBAT*_ at 0.75- (*P* < 0.05) and 1-h (*P* < 0.05) relative to vehicle treatment on day 24.

**TABLE 2:** T_IBAT_ measurements following acute systemic administration of the beta-3 receptor agonist (CL 316243) or vehicle in male DIO rats.

(A) IP injection day 1 (CL 316243; 0.001 mg/kg)									
Day 1	4V	IP	0 min	15 min	30 min	45 min	60 min	20 h	24 h

			Temp (C°)	Temp (C°)	Temp (C°)	Temp (C°)	Temp (C°)	Temp (C°)	Temp (C°)
	**VEH**	**VEH**	36.9 ± 0.2	37.6 ± 0.1	37.4 ± 0.2	37.1 ± 0.2	37.1 ± 0.1	37.1 ± 0.3	36.5 ± 0.1
	**VEH**	**CL 316243**	37.2 ± 0.2	38.6 ± 0.3*	38.6 ± 0.2*	38.3 ± 0.2*	38.2 ± 0.3*	37.0 ± 0.3	37.0 ± 0.2
	**OT**	**VEH**	37.6 ± 0.2	38.2 ± 0.3^†^	38.0 ± 0.3	38.2 ± 0.3*	38.1 ± 0.3*	37.4 ± 0.2	37.5 ± 0.3*
	**OT**	**CL 316243**	37.4 ± 0.2	38.7 ± 0.4*	38.8 ± 0.4*	38.5 ± 0.4*	38.3 ± 0.3*	37.1 ± 0.4	36.9 ± 0.3
**(B) IP injection day 24 (CL 316243; 0.5 mg/kg)**									
**Day 24**	**4V**	**IP**	**0 min**	**15 min**	**30 min**	**45 min**	**60 min**	**20 h**	**24 h**

			**Temp (C°)**	**Temp (C°)**	**Temp (C°)**	**Temp (C°)**	**Temp (C°)**	**Temp (C°)**	**Temp (C°)**

	**VEH**	**VEH**	36.6 ± 0.1	37.8 ± 0.2	37.4 ± 0.2	37.3 ± 0.1	37.2 ± 0.2	36.8 ± 0.2	36.5 ± 0.2
	**VEH**	**CL 316243**	37.4 ± 0.4	38.8 ± 0.3*	38.7 ± 0.3*	38.5 ± 0.3*	38.4 ± 0.3*	37.1 ± 0.5	37.1 ± 0.5
	**OT**	**VEH**	37.3 ± 0.2	38.3 ± 0.3	38.1 ± 0.3	38.2 ± 0.3*	38.1 ± 0.3*	37.1 ± 0.3	37.3 ± 0.3
	**OT**	**CL 316243**	36.7 ± 0.4	38.3 ± 0.4	38.3 ± 0.4	38.1 ± 0.4^†^	37.9 ± 0.4	36.6 ± 0.4	36.3 ± 0.5

(A) injection day 1 (0.001 mg/kg) and (B) injection day 24 (0.5 mg/kg). Data are expressed as mean ± SEM. *P < 0.05 vs. VEH; ^†^0.05 < *P* < 0.1 vs. VEH.

*N = 4–5/group.*

*Only N = 2–3/group for 24 h measurement on Day 24.*

CL 316243 in combination with OT also tended to elevate T_*IBAT*_ at 0.5- [*F*(1,7) = 6.081, P < 0.05], 0.75- [*F*(1,7) = 5.894, *P* < 0.05], and 1-h post-injection [*F*(1,7) = 4.881, *P* = 0.063], suggesting that increased BAT thermogenesis may also contribute to these effects. CL 316243 + OT increased T_*IBAT*_ at 0. 25-, 0. 5-, 0. 75-, and 1-h post-injection relative to vehicle treatment at 0. 25-, 0. 5-, 0. 75-, and 1-h (*P* < 0.05) on day 1. CL 316243 + OT also tended to increase T_*IBAT*_ at 0.75-h post-injection relative to vehicle treatment at 0.75-h (0.05 < *P* < 0.1) post-injection on day 24 (Tables 2A,B).

Overall, these findings suggest that 1 week treatment with lower doses of the beta-3 receptor agonist (CL 316243) do not appear to provide additional benefit to reduce body weight and body adiposity beyond that of OT alone and that more sustained treatment of higher doses of CL 316243 (0.5 mg/kg) are required in order to see additional benefits between the two treatments on body weight (see Study 3A).

### Gene Expression Data

We next determined the extent to which CL 316243 (0.5 mg/kg), OT (16 nmol/day), or the combination treatment increased thermogenic gene expression in IBAT relative to vehicle at 2-h post-CL 316243/vehicle injections.

Compared with VEH treatment, CL 316243 injections in both the CL 316243/Vehicle group and in the CL 316243/OT groups were accompanied by significant elevations of T_*IBAT*_ at 0.75 and 2-h post-injection (data not shown; *P* < 0.05).

#### IBAT

Consistent with published findings in mice and rats, chronic CL 316243 administration elevated relative levels of the thermogenic markers Gpr120 (Rosell et al., 2014; Suarez et al., 2014; Gonzalez-Hurtado et al., 2018), and DIO2 (de Jong et al., 2017; Table 3; *P* < 0.05). CL 316243 in combination with 4V OT also elevated Gpr120 (Rosell et al., 2014; Suarez et al., 2014; Gonzalez-Hurtado et al., 2018) and DIO2 (*P* < 0.05) but there was no difference in expression of either Gpr120 or DIO between CL 316243 in combination with 4V OT vs. CL 316343 alone (*P* = NS).

**TABLE 3:** Changes in IBAT gene expression following chronic 4V OT (16 nmol/day) and systemic CL 316243 (0.5 mg/kg) treatment in male DIO rats.

Treatment	VEH	4V OT	CL 316243	CL 316243 + 4V OT
**IBAT**				
*Adra2a*	1.0 ± 0.1^*a*^	2.1 ± 0.6^*a*^	2.5 ± 0.7^*a*^	1.6 ± 0.3^*a*^
*beta1ar*	1.0 ± 0.1^*a*^	1.1 ± 0.1^*a*^	1.0 ± 0.2^*a*^	1.0 ± 0.2^*a*^
*beta2ar*	1.0 ± 0.2^*a*^	1.3 ± 0.3^*a*^	1.0 ± 0.1^*a*^	0.9 ± 0.1^*a*^
*beta3ar*	1.0 ± 0.1^*a*^	1.3 ± 0.5^*a*^	0.6 ± 0.1^*a*^	0.5 ± 0.1^*a*^
*UCP1*	1.0 ± 0.2^*a*^	1.2 ± 0.2^*a*^	1.5 ± 0.4^*a*^	1.7 ± 0.3^*a*^
*UCP2*	1.0 ± 0.2^*a*^	1.1 ± 0.1^*a*^	1.3 ± 0.4^*a*^	1.8 ± 0.2^*a*^
*UCP3*	1.0 ± 0.1^*a*^	1.9 ± 0.5^*a*^	1.4 ± 0.6^*a*^	1.6 ± 0.3^*a*^
*bmp8b*	1.0 ± 0.5^*a*^	1.0 ± 0.2^*a*^	1.7 ± 0.8^*a*^	1.2 ± 0.1^*a*^
*Cidea*	1.0 ± 0.2^*a*^	1.4 ± 0.4^*a*^	1.7 ± 0.5^*a*^	1.2 ± 0.3^*a*^
*Gpr120*	1.0 ± 0.1^*a*^	1.7 ± 0.3^*a*^	16.1 ± 3.7^*b*^	10.7 ± 2.1^*b*^
*Cox8b*	1.0 ± 0.2^*a*^	1.3 ± 0.2^*a*^	1.4 ± 0.1^*a*^	0.9 ± 0.1^*a*^
*DIO2*	1.0 ± 0.1^*a*^	2.1 ± 0.3^*ac*^	4.2 ± 1.1^*b*^	3.6 ± 0.7^*bc*^
*Ppargc1a*	1.0 ± 0.1^*a*^	1.4 ± 0.3^*a*^	13.8 ± 7.1^*a*^	8.1 ± 2.7^*a*^

*Data are expressed as mean ± SEM. Different letters denote significant differences between treatments (*P* < 0.05).*

*Shared letters are not significantly different from one another.*

*N = 4–5/group.*

### Study 3: Effect of Chronic 4V OT Infusions (16 nmol/day) and Systemic Beta-3 Receptor Agonist (CL 31643) Administration (0.5 mg/kg) on Body Weight, Body Adiposity and Energy Intake in Male DIO Rats

The goal of this study was to determine the effects of chronic OT treatment in combination with a single dose (identified in Study 2A) of the beta-3 receptor agonist, CL 316243, on body weight and adiposity in DIO rats. By design, DIO rats were obese as determined by both body weight (804 ± 14 g) and adiposity (310 ± 11 g fat mass; 38.3 ± 4.7% adiposity) after maintenance on the HFD for approximately 7 months. Prior to the onset of CL 316243 treatment on infusion day 7, both OT treatment groups were matched for OT-elicited reductions of weight gain.

**FIGURE 3:**
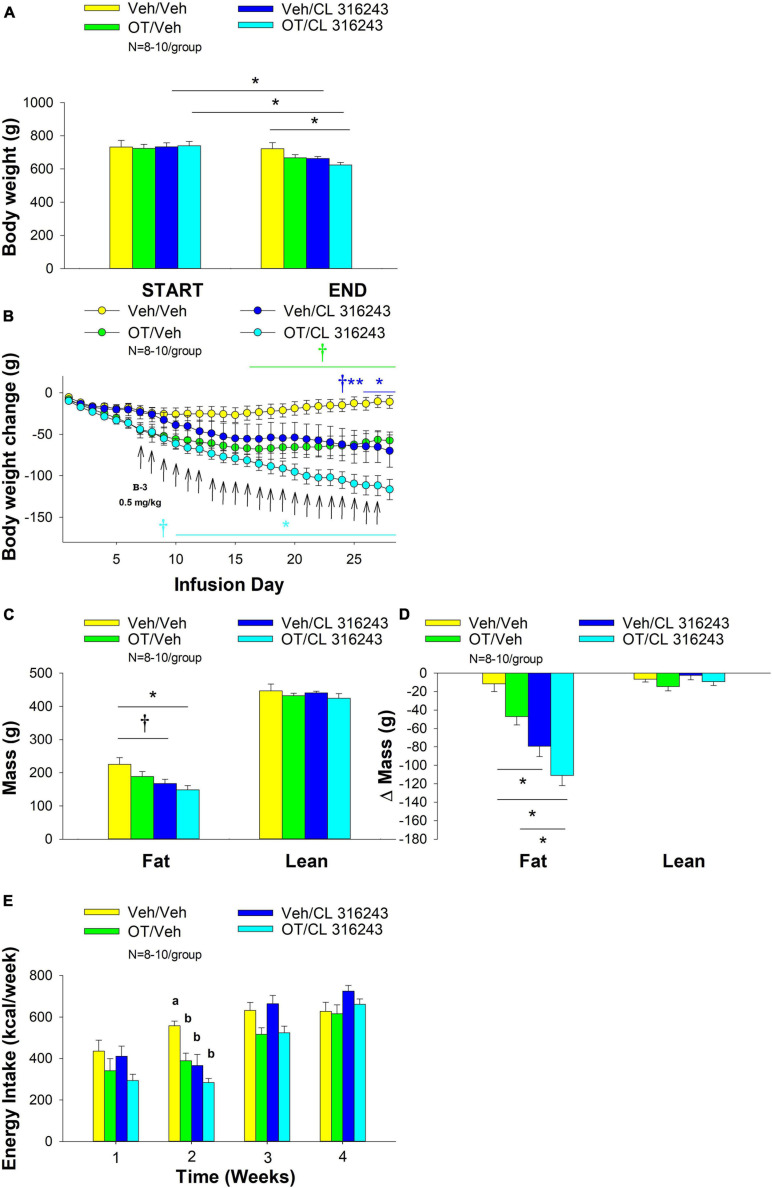
**(A–E)** Effect of chronic 4V OT infusions (16 nmol/day) and systemic beta-3 receptor agonist (CL 31643) administration (0.5 mg/kg) on body weight, body adiposity and energy intake in male DIO rats. *Ad libitum* fed rats were maintained on HFD (60% kcal from fat; *N* = 8–10/group) for approximately 8 months prior to receiving continuous infusions of vehicle or OT (16 nmol/day) in combination with a single dose of CL 316243 (0.5 mg/kg). **(A)** Change in body weight in HFD-fed DIO rats; **(B)** Change in body weight gain in HFD-fed DIO rats; **(C)** Change in fat mass and lean mass in HFD-fed DIO rats; **(D)** Change in relative fat mass and lean mass in HFD-fed DIO rats; **(E)** Weekly energy intake (kcal/week) in HFD-fed DIO rats. ↑ indicate 1× daily injections. Different letters denote significant differences between treatments. **(B)** Colored bars represent specific group comparisons vs. vehicle. Data are expressed as mean ± SEM. *P < 0.05, **P = 0.05, ^†^0.05 < *P* < 0.1 vs. vehicle or baseline (pre-treatment; **A**).

Oxytocin and CL 316243 alone reduced body weight by ≈ 7.8 ± 1.3% (*P* < 0.05) and 9.1 ± 2.3% (*P* < 0.05), respectively, but the combined treatment produced more pronounced weight loss (pre- vs. post-intervention) (15.5 ± 1.2%; *P* < 0.05) (Figure 3A) than either treatment alone (*P* < 0.05). OT alone tended to reduce weight gain on days 16–28 (0.05 < *P* < 0.1) while CL 316243 alone tended to reduce or reduced weight gain on day 24 (0.05 < *P* < 0.1), day 25 (*P* = 0.05), and days 26–28 (*P* < 0.05) (Figure 3B). OT and CL 316243 together tended to reduce weight gain on day 9 (0.05 < *P* < 0.1) reduced weight gain on days 10–28 (*P* < 0.05). The combination treatment appeared to produce a more pronounced reduction of weight gain relative to OT alone on day 25 (0.05 < *P* < 0.1) and this reached significance on days 26–28 (*P* < 0.05). In addition, the combination treatment tended to produce a greater reduction of weight gain relative to CL 316243 alone on days 21–22 and 26–28 (0.05 < *P* < 0.1). While OT alone did not significantly reduce fat mass (*P* = NS), there was a tendency for CL 316243 alone (0.05 < *P* < 0.1), and the combination of OT and CL 316243 (*P* < 0.05) to reduce fat mass without impacting lean body mass (Figure 3C; *P* = NS). However, the combination treatment did not result in a significant reduction of fat mass relative to OT alone or CL 316243 alone (*P* = NS). Consistent with these effects, OT alone did not produce a reduction in relative fat mass (*P* = 0.108) while CL 316243 alone reduced relative fat mass (pre vs. post-intervention; *P* < 0.05) without changing lean mass (Figure 3D; *P* = NS). The combination treatment produced a significant reduction of relative fat mass (pre vs. post-intervention; *P* < 0.05) which exceeded that of OT alone (*P* < 0.05) but not CL 316243 alone (*P* = NS). OT, CL 316243 and the combined treatment were all effective at reducing energy intake at week 2 (Figure 3E; *P* < 0.05) with no difference between treatments (*P* = NS). All treatments were ineffective at reducing energy intake over weeks 3 and 4 (*P* = NS).

Two-way ANOVA failed to reveal a significant interactive effect between OT and CL 316243 on reduction of weight loss, change in daily body weight, fat mass or energy intake. These findings suggest that OT does not interact with CL 316243 to impact body weight, adiposity or energy intake at the doses used in the current study (Seeley and Moran, 2002).

We initially confirmed that there was no difference in T_*IBAT*_ at baseline (0; immediately prior to injections) on IP injection days 1 or 19 (Tables 4A,B). In addition, repeated measures ANOVA (IP injection days 1, 19) indicated there tended to be a significant or near significant main effect of CL 316243 alone to elevate T_*IBAT*_ throughout the IP injection period at 0.25- [*F*(1,15) = 3.893, *P* = 0.067], 0.5- [*F*(1,15) = 4.520, *P* = 0.051], 0.75- [*F*(1,15) = 7.525, *P* < 0.05], 1- [*F*(1,15) = 6.754, *P* < 0.05], and at 20-h post-injection [*F*(1,14) = 4.188, *P* = 0.060]. CL 316243 increased T_*IBAT*_ at 0. 25-, 0. 5-, 0. 75-, and 1-h post-injection relative to vehicle treatment at 0.25- (*P* = 0.07), 0.5- (*P* = 0.078), 0.75- (*P* < 0.05), 1- (*P* < 0.05), and 20-h post-injection (*P* < 0.05) on day 1. Similarly, CL 316243 also increased T_*IBAT*_ at 0. 25-, 0. 5-, 0. 75-, and 1-h post-injection relative to vehicle treatment at 0.25- (*P* = 0.093), 0.5- (*P* < 0.05), 0.75- (*P* < 0.05), and 1-h (*P* = 0.054) on day 19 (Tables 4A,B). In contrast to Study 2, repeated measures ANOVA (IP injection days 1, 19) indicated there was not a significant main effect of OT to elevate T_*IBAT*_ (Tables 4A,B).

**TABLE 4:** T_IBAT_ measurements following acute systemic administration of the beta-3 receptor agonist (CL 316243) or vehicle in male DIO rats.

(A) IP injection day 1 (CL 316243; 0.5 mg/kg)									
Day 1	4V	IP	0 min	15 min	30 min	45 min	60 min	20 h	24 h

			Temp (C°)	Temp (C°)	Temp (C°)	Temp (C°)	Temp (C°)	Temp (C°)	Temp (C°)
	**VEH**	**VEH**	37.1 ± 0.3	37.8 ± 0.3	37.7 ± 0.2	37.4 ± 0.2	37.4 ± 0.2	36.4 ± 0.2	36.9 ± 0.2
	**VEH**	**CL 316243**	36.9 ± 0.3	38.5 ± 0.2^†^	38.3 ± 0.2^†^	38.2 ± 0.2*	38.2 ± 0.2*	37.5 ± 0.3*	37.5 ± 0.3
	**OT**	**VEH**	37.0 ± 0.2	37.8 ± 0.2	37.5 ± 0.2	37.4 ± 0.2	37.5 ± 0.2	36.5 ± 0.2	36.9 ± 0.3
	**OT**	**CL 316243**	37.3 ± 0.1	38.9 ± 0.2	38.6 ± 0.2	38.3 ± 0.2	38.2 ± 0.2	37.7 ± 0.3*	37.7 ± 0.2

**(B) IP injection day 19 (CL 316243; 0.5 mg/kg)**									

**Day 19**	**4V**	**IP**	**0 min**	**15 min**	**30 min**	**45 min**	**60 min**	**20 h**	**24 h**

			**Temp (C°)**	**Temp (C°)**	**Temp (C°)**	**Temp (C°)**	**Temp (C°)**	**Temp (C°)**	**Temp (C°)**

	**VEH**	**VEH**	37.1 ± 0.2	37.7 ± 0.2	37.5 ± 0.2	37.4 ± 0.3	37.3 ± 0.3	36.6 ± 0.6	36.9 ± 0.3
	**VEH**	**CL 316243**	36.7 ± 0.2	38.3 ± 0.3^†^	38.2 ± 0.2*	38.2 ± 0.2*	38.1 ± 0.2^†^	36.7 ± 0.2	37.0 ± 0.2
	**OT**	**VEH**	36.7 ± 0.2	37.7 ± 0.1	37.5 ± 0.1	37.4 ± 0.1	37.4 ± 0.2	36.5 ± 0.5	36.8 ± 0.2
	**OT**	**CL 316243**	36.9 ± 0.2	38.5 ± 0.3*	38.4 ± 0.3*	38.3 ± 0.3*	38.2 ± 0.3*	36.9 ± 0.2	36.9 ± 0.3

(A) injection day 1 (0.5 mg/kg) and (B) injection day 19 (0.5 mg/kg). Data are expressed as mean ± SEM. *P < 0.05 vs. VEH; ^†^0.05 < *P* < 0.1 vs. VEH.

**N* = 8–10/group.*

Similarly, there tended to be a significant main effect of CL 316243 in combination with OT to elevate T_*IBAT*_ at 0.25- [*F*(1,17) = 7.815, *P* < 0.05], 0.5- [*F*(1,17) = 9.970, *P* < 0.05], 0.75- [*F*(1,17) = 10.599, *P* < 0.05], 1- [*F*(1,17) = 7.567, *P* < 0.05], and 20-h post-injection [*F*(1,16) = 7.427, *P* < 0.05], suggesting that increased BAT thermogenesis may also contribute to these effects. CL 316243 + OT increased T_*IBAT*_ at 0. 25-, 0. 5-, 0. 75-, 1-, and 20-h post-injection relative to vehicle treatment at 0. 25-, 0. 5-, 0. 75-, 1-, and 20-h (*P* < 0.05) on day 1. CL 316243 + OT also increased T_*IBAT*_ at 0. 25-, 0. 5-, 0. 75-, and 1-h post-injection relative to vehicle treatment at 0. 25-, 0. 5-, 0. 75-, and 1-h (*P* < 0.05) on day 19 (Tables 4A,B).

Overall, these findings suggest an additive effect of OT and CL 316243 to produce sustained weight loss in DIO rats.

### Adipocyte Size

**FIGURE 4:**
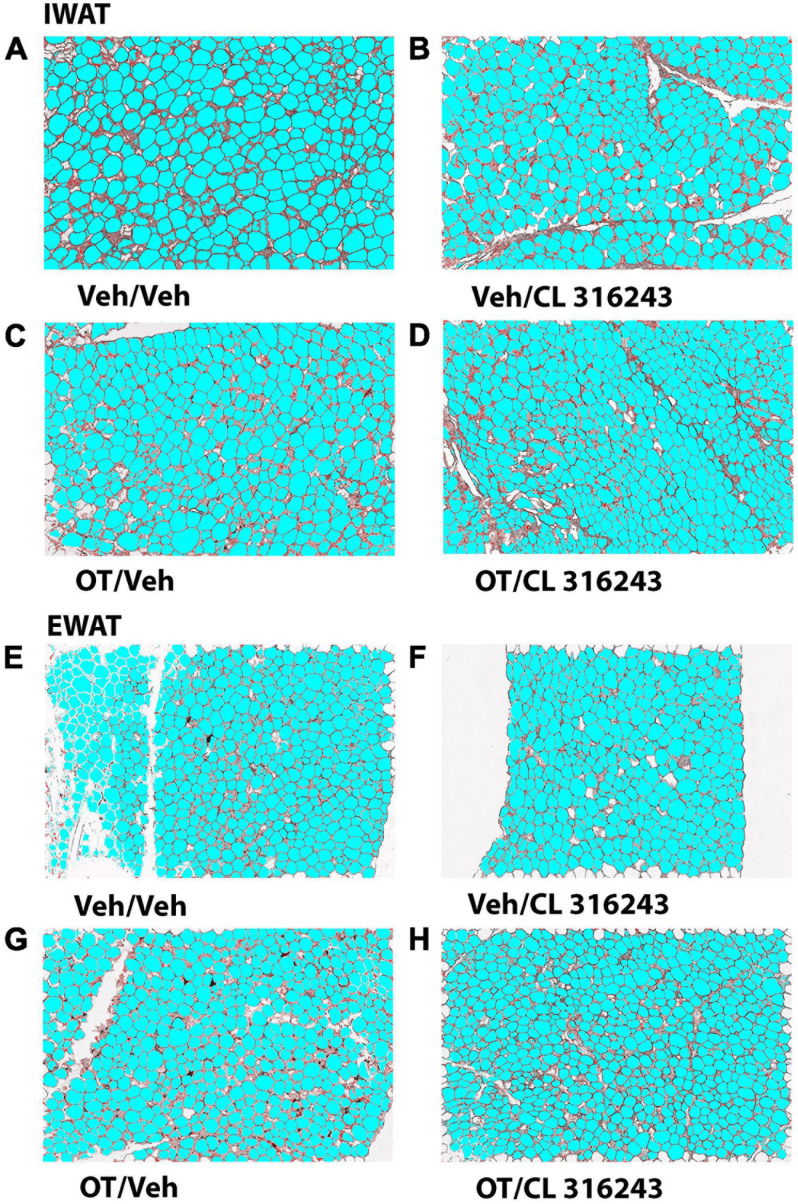
**(A–H)** Effect of chronic 4V OT infusions (16 nmol/day) and systemic beta-3 receptor agonist (CL 31643) administration (0.5 mg/kg) on adipocyte size in IWAT and EWAT in male DIO rats. Adipocyte size was analyzed using ImageJ. Images were taken from fixed (4% PFA) paraffin embedded sections (5 μm) containing IWAT **(A–D)** or EWAT **(E–H)** in DIO rats treated with 4V OT (16 nmol/day) or 4V vehicle in combination with IP CL 316243 (0.5 mg/kg) or IP vehicle. A/E, Veh/Veh; B/F, Veh/CL 316243; C/G, OT/Veh; D/H, OT-CL 316243; **(A–H)** all visualized at 100× magnification.

**FIGURE 5:**
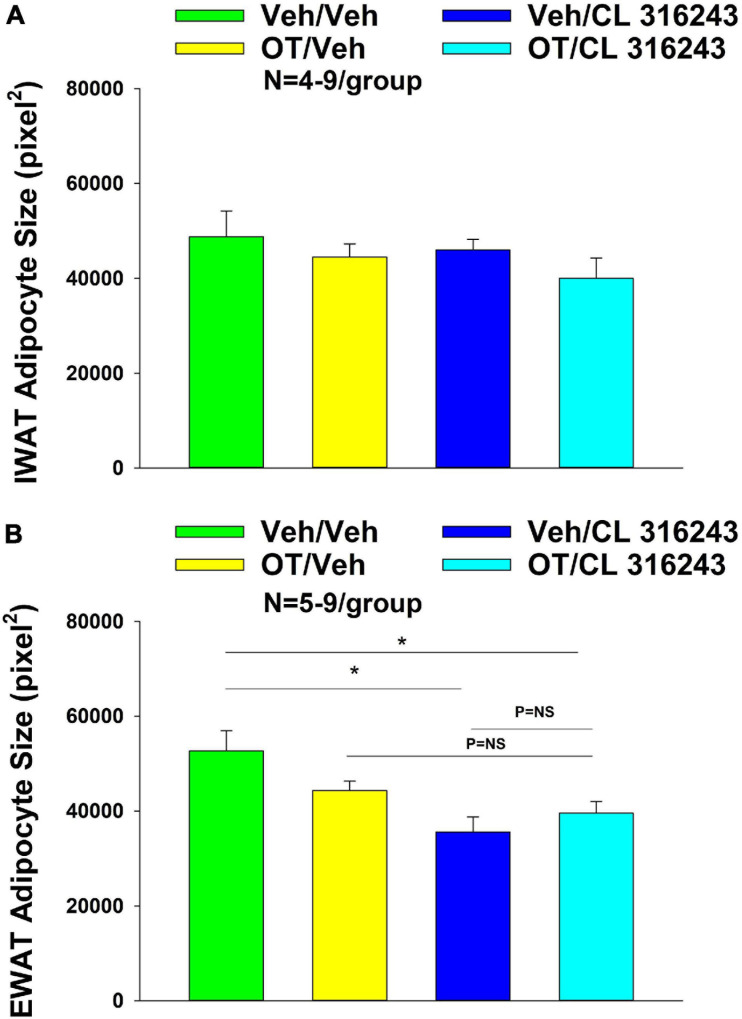
**(A,B)** Effect of chronic 4V OT infusions (16 nmol/day) and systemic beta-3 receptor agonist (CL 31643) administration (0.5 mg/kg) on adipocyte size in IWAT and EWAT in male DIO rats. **(A)** Adipocyte size (pixel^2^) was measured in IWAT from rats that received chronic 4V infusion of OT (16 nmol/day) or vehicle in combination with daily CL 316243 (0.5 mg/kg) or vehicle treatment (*N* = 4–9/group). **(B)** Adipocyte size was measured in EWAT from rats that received chronic 4V infusion of OT (16 nmol/day) or vehicle in combination with daily CL 316243 (0.5 mg/kg) or vehicle treatment (*N* = 5–9/group). Data are expressed as mean ± SEM. *P < 0.05 OT vs. vehicle.

Oxytocin and CL 316243 given alone or in combination failed to significantly impact IWAT adipocyte size in DIO rats (*P* = NS) (Figures 4A–D, 5A). In contrast, CL 316243 alone (*P* < 0.05) or in combination with OT (*P* < 0.05) reduced EWAT adipocyte size in DIO rats relative to vehicle treatment (Figures 4E–H, 5B). There were no significant differences in the ability of the combined treatment to reduce EWAT adipocyte size relative to OT alone or CL 316243 alone (*P* = NS). In addition, there were no significant differences on EWAT adipocyte size between OT alone and CL 316243 alone (*P* = NS).

### UCP-1 Expression

**FIGURE 6:**
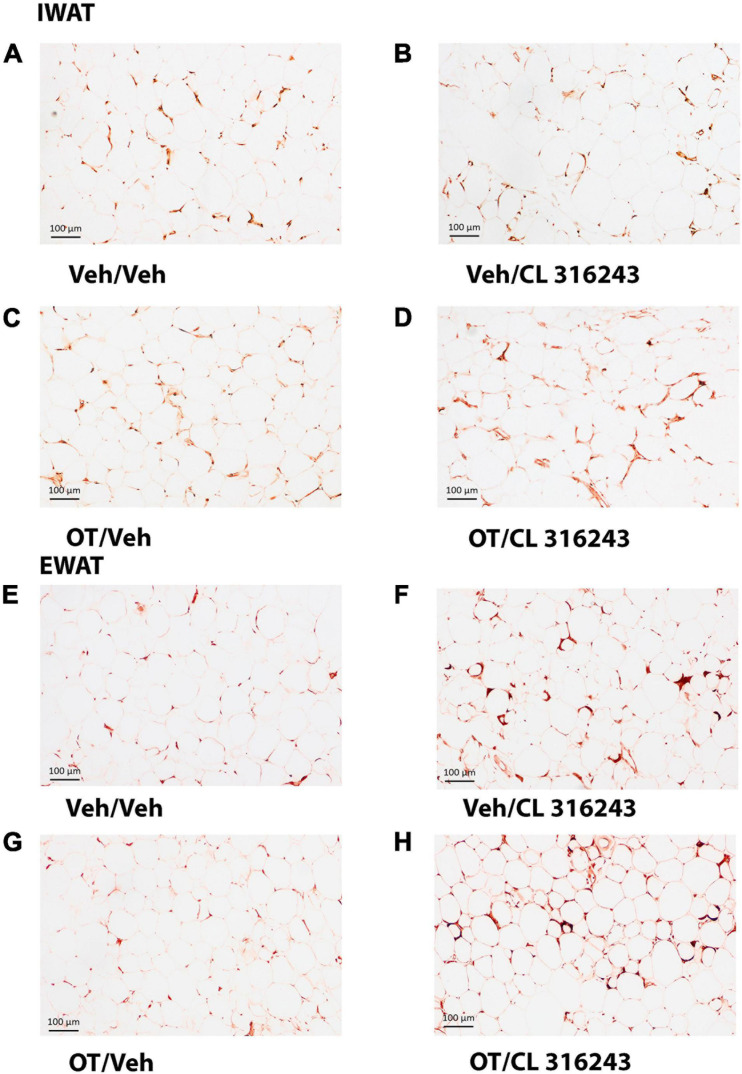
**(A–H)** Effect of chronic 4V OT infusions (16 nmol/day) and systemic beta-3 receptor agonist (CL 31643) administration (0.5 mg/kg) on UCP-1 content in IWAT and EWAT in male DIO rats. UCP-1 was analyzed using Image Pro Plus software. Images were taken from fixed (4% PFA) paraffin embedded sections (5 μm) containing IWAT **(A–D)** or EWAT **(E–H)** in DIO rats treated with 4V OT (16 nmol/day) or 4V vehicle in combination with IP CL 316243 (0.5 mg/kg) or IP vehicle. A/E, Veh/Veh; B/F, Veh/CL 316243; C/G, OT/Veh; D/H, OT/CL 316243; **(A–H)** all visualized at 100× magnification.

**FIGURE 7:**
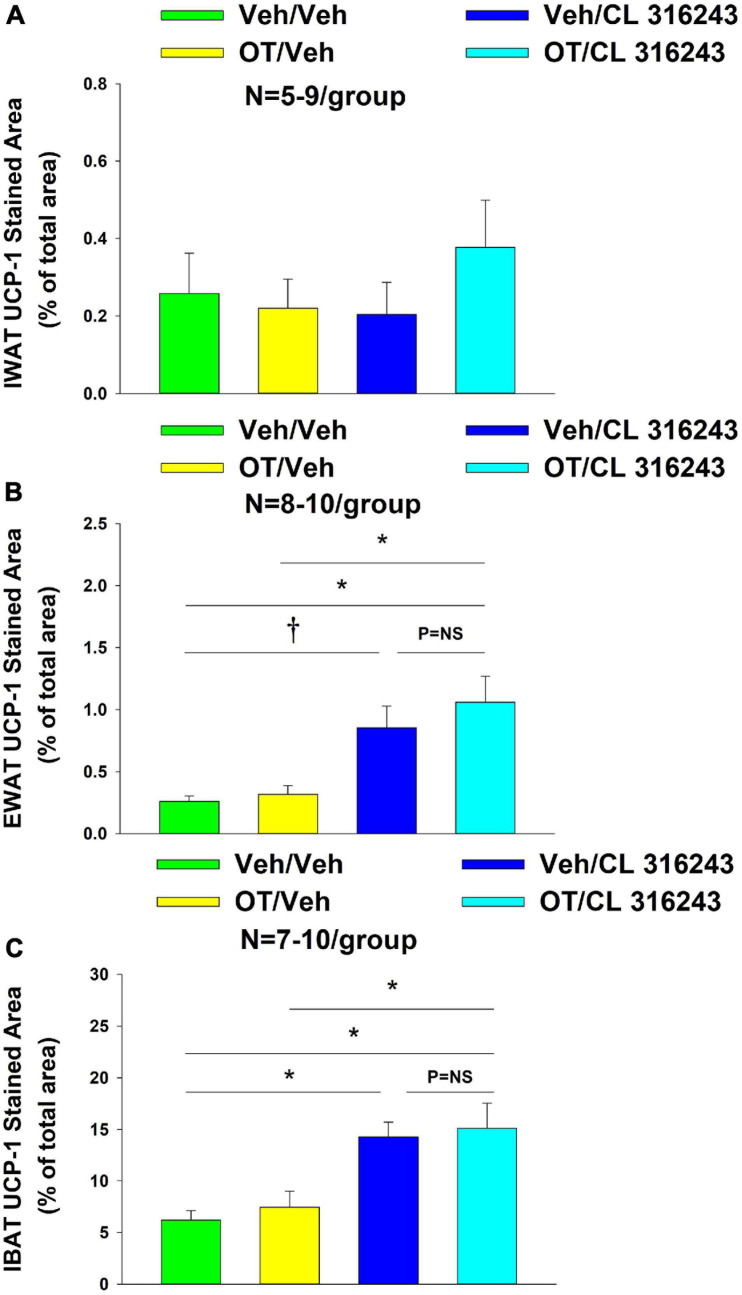
**(A–C)** Effect of chronic 4V OT infusions (16 nmol/day) and systemic beta-3 receptor agonist (CL 31643) administration (0.5 mg/kg) on UCP-1 content in IWAT, EWAT, and IBAT in male DIO rats. UCP-1 was analyzed from IWAT, EWAT, and IBAT **(A–C)** in DIO rats treated with 4V OT (16 nmol/day) or 4V vehicle in combination with IP CL 316243 (0.5 mg/kg) or IP vehicle. **(A)** IWAT. **(B)** EWAT. **(C)** IBAT. Data are expressed as mean ± SEM. *P < 0.05, ^†^0.05 < *P* < 0.1.

Oxytocin and the beta-3 agonist alone as well as OT and CL 316243 together failed to increase UCP-1 expression in IWAT relative to VEH (*P* = NS) (Figures 6A–D, 7A).

**FIGURE 8:**
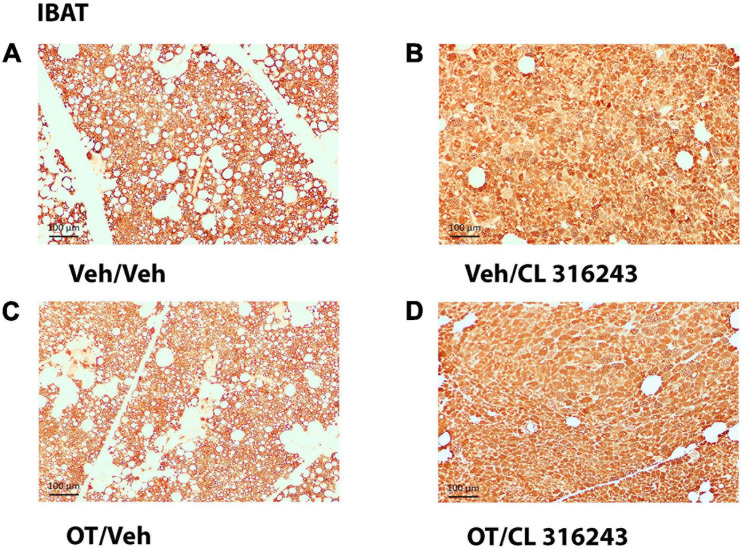
**(A–D)** Effect of chronic 4V OT infusions (16 nmol/day) and systemic beta-3 receptor agonist (CL 31643) administration (0.5 mg/kg) on UCP-1 content in IBAT in male DIO rats. UCP-1 staining was quantified in IBAT from DIO rats that received chronic 4V infusion of OT (16 nmol/day) or vehicle in combination with daily CL 316243 (0.5 mg/kg) or vehicle treatment (*N* = 7–10/group). **(A)** Veh/Veh. **(B)** Veh/CL 316243. **(C)** OT/Veh. **(D)** OT/CL 316243. **(A–D)** All visualized at 100× magnification.

Additionally, OT (*P* = NS) alone failed to increase UCP-1 content in EWAT. However, CL 316243 alone (*P* = 0.052) and in combination with OT (*P* < 0.05) increased UCP-1 expression in EWAT relative to VEH. The combination of CL 316243 and OT also increased UCP-1 relative to OT treatment alone (Figures 6E–H, 7B) but was not different from CL 316243 alone (*P* = NS). There was also no significant difference between CL 316243 and OT when given alone (*P* = 0.102). CL316243 in combination with OT also increased UCP-1 expression in IBAT relative to VEH and OT treatment alone (*P* < 0.05; Figures 8A–D, 7C) but there was no significant difference relative to CL 316243 alone (*P* = NS). CL 316243 alone stimulated IBAT UCP-1 relative to VEH alone (*P* < 0.05) and tended to stimulate IBAT UCP-1 relative to OT alone (*P* = 0.087). OT (*P* = NS) failed to significantly increase UCP-1 in IBAT relative to VEH.

### Plasma Hormone Concentrations

To characterize the endocrine and metabolic effects of 4V OT (16 nmol/day) and systemic beta-3 receptor agonist (CL 316243) in DIO rats in both the dose-escalation study (Study 2; Table 5) and chronic study using as single dose of CL 316243 (Study 3; Table 6), we measured blood glucose levels and plasma concentrations of leptin, insulin, FGF-21, irisin, adiponectin, TC, and FFAs. The combination treatment in Study 2 was associated with an increase of FFA relative to vehicle treated animals (*P* < 0.05) but it was not significantly different from CL 316243 alone (*P* = NS) or OT alone (*P* = 0.097). CL 316243 alone tended to reduce plasma TC relative to vehicle (*P* = 0.080). The combination treatment of a single dose in Study 3 was associated with a reduction of plasma leptin relative to VEH in DIO rats in Study 3 in which fat mass was also reduced. CL 316243 and OT treatment also reduced plasma leptin relative to OT alone (*P* < 0.05). In addition, in Study 3, the combination treatment also reduced blood glucose and elevated adiponectin levels. In contrast, OT, CL 316243 or the combination treatment failed to alter plasma concentrations of total cholesterol, insulin, irisin, FGF-21.

**TABLE 5:** Plasma measurements following 4V infusions of OT (16 nmol/day), CL 316243 (0.5 mg/kg), or OT (16 nmol/day) + CL 316243 (0.5 mg/kg) in DIO rats.

4V Treatment	Vehicle	OT	CL 316243	OT + CL 316243
	
	HFD	HFD	HFD	HFD
**Leptin (ng/mL)**	36.8 ± 6.9^*a*^	25.9 ± 2.5^*a*^	26.7 ± 2.6^*a*^	20.9 ± 3.1^*a*^
**Insulin (ng/mL)**	1.7 ± 0.3^*a*^	1.0 ± 0.2^*a*^	2.1 ± 0.6^*a*^	1.4 ± 0.2^*a*^
**FGF-21 (pg/mL)**	140.8 ± 35^*a*^	134 ± 21.6^*a*^	59.3 ± 17.2^*a*^	131.2 ± 38.1^*a*^
**Irisin (μg/mL)**	5.3 ± 0.6^*a*^	6.2 ± 1.4^*a*^	5.6 ± 0.4^*a*^	5.2 ± 0.6^*a*^
**Adiponectin (μg/mL)**	7.4 ± 0.9^*a*^	6.7 ± 0.5^*a*^	10.3 ± 1.4^*a*^	8.5 ± 1.2^*a*^
**Blood Glucose (mg/dL)**	156.8 ± 9.8^*a*^	153 ± 10.1^*a*^	129.5 ± 7.5^*a*^	132.3 ± 8.2^*a*^
**FFA (mEq/L)**	0.24 ± 0.02^*a*^	0.27 ± 0.07^*ab*^	0.34 ± 0.07^*ab*^	0.48 ± 0.04^*b*^
**Total Cholesterol (mg/dL)**	102.8 ± 4.6^*a*^	95.0 ± 4.0^*ab*^	85.6 ± 3.1^*b*^	96.9 ± 5.1^*ab*^

*Different letters denote significant differences between treatments.*

*Shared letters are not significantly different from one another.*

*N = 4–5/group.*

**TABLE 6:** Plasma measurements following 4V infusions of OT (16 nmol/day), CL 316243 (0.5 mg/kg), or OT (16 nmol/day) + CL 316243 (0.5 mg/kg) in DIO rats.

4V Treatment	Vehicle	OT	CL 316243	OT + CL 316243
	
	HFD	HFD	HFD	HFD
**Leptin (ng/mL)**	39.4 ± 6.3^*a*^	38.5 ± 4.3^*a*^	24.9 ± 3.4^*ab*^	20.4 ± 3.6^*b*^
**Insulin (ng/mL)**	2.0 ± 0.3^*a*^	2.2 ± 0.3^*a*^	1.3 ± 0.2^*a*^	3.2 ± 1.4^*a*^
**FGF-21 (pg/mL)**	146.4 ± 17.8^*a*^	142.3 ± 12.5^*a*^	128.7 ± 27^*a*^	174.9 ± 68.4^*a*^
**Irisin (μg/mL)**	5.5 ± 1.0^*a*^	5.6 ± 0.9^*a*^	5.4 ± 0.7^*a*^	5.5 ± 0.5^*a*^
**Adiponectin (μg/mL)**	7.2 ± 0.4^*a*^	7.4 ± 0.6^*a*^	9.0 ± 0.5^*ab*^	11.1 ± 1.1^*b*^
**Blood Glucose (mg/dL)**	161.5 ± 7.8^*a*^	153.9 ± 4.8^*a*^	150.7 ± 8.1^*a*^	141 ± 5.0^*a*^
**FFA (mEq/L)**	0.25 ± 0.02^*a*^	0.28 ± 0.06^*a*^	0.32 ± 0.05^*a*^	0.28 ± 0.03^*a*^
**Total Cholesterol (mg/dL)**	93.9 ± 8.4^*a*^	95.9 ± 3.5^*a*^	104.9 ± 6.4^*a*^	106.8 ± 7.7^*a*^

*Different letters denote significant differences between treatments.*

*Shared letters are not significantly different from one another.*

**N* = 8–10/group.*

## Discussion

The goal of the current studies was to determine if OT can be used as an adjunct with other drugs that directly target beta-3 receptors in BAT to reduce body weight in DIO rats. We hypothesized that the combined treatment of OT and the beta-3 agonist, CL 316243, would produce an additive effect to decrease body weight and adiposity in DIO rats by reducing energy intake and increasing BAT thermogenesis and browning of EWAT. To address this, we assessed the effects of 4V infusions of OT (16 nmol/day) or vehicle (VEH) in combination with daily IP injections of CL 316243 (0.5 mg/kg) or VEH on food intake, interscapular BAT temperature (T_*IBAT*_), body weight and body composition. OT and CL 316243 alone reduced body weight by 7.9% (0.05 < *P* < 0.1) and 9.6% (*P* < 0.05), respectively, but the combined treatment produced more pronounced weight loss (15.7%; *P* < 0.05). These effects were associated with decreases in energy intake, adiposity, EWAT adipocyte size and browning of EWAT (*P* < 0.05). During treatment, CL 316243 alone stimulated T_*IBAT*_ at 0.25, 0.5 (*P* = 0.051), 0.75 and 1-h post-injection (*P* < 0.05). Similarly, the combination of OT and CL 316243 resulted in elevations of T_*IBAT*_ at 0.25, 0.5, 0.75, and 1-h post-injection (*P* < 0.05) relative to VEH throughout the course of the IP injection study. In addition, the combination treatment elevated IBAT UCP-1 content and elevated IBAT thermogenic gene expression suggesting that increased BAT thermogenesis may also contribute to these effects. These findings are consistent with the hypothesis that the combined treatment of OT and the beta-3 agonist, CL 316243, produces an additive effect to decrease body weight. The findings from the current study suggest that the effects of the combined treatment on energy intake, fat mass, adipocyte size and browning of EWAT were not additive and appear to be driven, in part, by transient changes in energy intake in response to OT or CL 316243 alone as well as CL 316243-elicited reduction of fat mass and adipocyte size and induction of browning of EWAT.

Our findings are consistent with recent data showing that the fat-induced satiety signal and peroxisome proliferator-activating receptor-α (PPARα) agonist, oleoylethanolamide (OEA) in combination with CL 316243 produced an additive effect to reduce food intake and weight gain. In contrast, OEA and CL 316243 produced what appeared to be a synergistic effect to reduce fat mass and increase expression of the thermogenic markers PPARα and UCP-1 expression in EWAT (Suarez et al., 2014). These findings are particularly interesting given that Deblon and colleagues found that both chronic central and systemic infusions of OT elevated OEA expression in EWAT (Deblon et al., 2011). Furthermore, Deblon and colleagues found that OT’s ability to reduce body weight was attenuated in PPARα null mice (Deblon et al., 2011) suggesting that PPARα signaling contributes, in part to OT’s metabolic effects on EWAT. Interestingly, recent findings indicate that OEA also increases OT mRNA expression in the hypothalamus (Gaetani et al., 2010), activates PVN OT neurons (Romano et al., 2013), stimulates the release of OT within the PVN (Romano et al., 2013), and reduces food intake, in part, through an OT receptor-dependent mechanism (Gaetani et al., 2010). Collectively, these findings suggest that OEA released in response to a high fat diet (Sospedra et al., 2015) may suppress food intake, in part, through an OT-dependent mechanism and that OT’s thermogenic effects within WAT may occur, in part, through a PPARα-dependent mechanism. Further studies using tissue specific ablation of PPARα will be helpful in determining the extent to which PPARα within this fat depot contribute to the additive effects of OT and CL 316243 on body weight and fat mass.

Our finding that OT produced these effects when given into the hindbrain (4V) suggest that hindbrain populations and/or spinal cord populations may contribute to the additive effects of OT on CL-316423-elicited reductions of body weight in a rat model. Recent anatomical data indicate the existence of both shared and separate CNS circuits that control SNS outflow to IBAT and IWAT (Nguyen et al., 2016). In particular, parvocellular PVN OT neuronal cell bodies have been found to project to IBAT (Oldfield et al., 2002; Doslikova et al., 2019), EWAT (Shi and Bartness, 2001; Stanley et al., 2010) and IWAT (Shi and Bartness, 2001; Doslikova et al., 2019) and a small subset of PVN OT neurons overlap and project to both IBAT and IWAT (Doslikova et al., 2019). OT neurons are thus anatomically positioned to control SNS outflow to IBAT and IWAT to regulate fat mass and adipocyte size. It remains to be determined whether these functions occur through either the same subset of neurons or adjacent OT neurons within the parvocellular PVN. This could occur, in part, through direct descending projections to the hindbrain NTS (Sawchenko and Swanson, 1982; Rinaman, 1998) and/or spinal cord (Sawchenko and Swanson, 1982), both of which may regulate sympathetic nervous system outflow to IBAT and are linked to the control of BAT thermogenesis (Bamshad et al., 1999; Cano et al., 2003). Whether OT acts at OTRs within the NTS, other hindbrain areas (including the raphe pallidus) (Cano et al., 2003; Morrison and Nakamura, 2011; Kong et al., 2012; Kasahara et al., 2015; Morrison, 2016) or spinal cord (Sutton et al., 2014) contribute to OT-induced reductions of fat mass and adipocyte size remains to be determined.

It is not clear why the combined treatment and CL 316243 elicited differential effects on browning in IWAT and EWAT although others have identified that certain stimuli may differentially modulate SNS outflow to fat depots. Bartness and colleagues have found that central administration of melanotan II (MTII), which may act, in part through OT signaling (Olszewski et al., 2001; Liu et al., 2003; Yosten and Samson, 2010; Modi et al., 2015; Semple et al., 2019), elicits differential effects on SNS outflow to WAT depots (Brito et al., 2007). MTII increased norepinephrine turnover (NETO; readout of SNS outflow) to inguinal WAT as well as dorsosubcutaneous WAT and IBAT NETO, but failed to impact SNS outflow to EWAT or retroperitoneal WAT. Our findings raise the possibility that there are distinct projections originating from OT receptor populations within CNS sites including the NTS or raphe pallidus that regulate SNS outflow to WAT (Shi and Bartness, 2001; Song et al., 2009; Stanley et al., 2010; Adler et al., 2012) and express OT receptors [mice: (Gould and Zingg, 2003; Yoshida et al., 2009)/rats: (Verbalis et al., 1995; Baskin et al., 2010; Ong et al., 2015, 2017)].

While our study also implicates a role for hindbrain and/or spinal cord OT receptors in the control of adipocyte size in a rat model, others have found similar effects following peripheral administration suggesting that these effects may also occur through a direct mechanism. Subcutaneous infusion of OT was found to reduce adipocyte size in subcutaneous fat in female Wistar rats that were ovariectomized (Iwasa et al., 2019) (≈ 496 nmol/day) EWAT of fatty Zucker rats (Balazova et al., 2016) (≈ 12.5 nmol/day), in visceral fat (which included parametrial, perirenal, and mesenteric depots) in peri- and postmenopausal female Wistar rats (Erdenebayar et al., 2020) (≈ 992.8 nmol/day) and in visceral fat in female Wistar rats in a model of polycystic ovary syndrome (Iwasa et al., 2020) (≈ 377 nmol/day). While it is possible that the effects observed on fat mass and adiposity size in our study may be attributed, in part, to leakage from the 4V to OT receptors in the spinal cord and/or periphery it is important to note that, with the exception of the one study in fatty Zucker rats (Balazova et al., 2016), the majority of doses used in the earlier studies in Wistar rats were approximately 23 to 62-fold higher than that found to be effective following hindbrain delivery in the current study. Collectively, these findings suggest that, in addition to a central mechanism mediated through hindbrain and/or spinal cord OTRs, OT may also act peripherally to reduce adipocyte size through a direct action on OTRs found on adipocytes (Schaffler et al., 2005; Deblon et al., 2011; Yi et al., 2015). Of translational relevance is the observation that systemic [intraperitoneal (Morton et al., 2012) or subcutaneous (Deblon et al., 2011; Maejima et al., 2011, 2017; Altirriba et al., 2014; Blevins et al., 2016)] administration of OT can recapitulate the effects of chronic CNS administration of OT on reductions of fat mass, adipocyte size, food intake and/or weight loss.

Our finding that the combination treatment and CL 316243 resulted in elevations of T_*IBAT*_ throughout the course of the IP injection study, IBAT UCP-1 content and elevated IBAT thermogenic genes (Gpr120 and DIO) suggest that increased BAT thermogenesis is one mechanism that contributes to the reduction of body weight and adiposity. These findings are in agreement with previous published findings in mice and rats that found chronic CL 316243 administration to elevate the thermogenic markers Gpr120 (Rosell et al., 2014; Suarez et al., 2014; Gonzalez-Hurtado et al., 2018) and DIO2 (de Jong et al., 2017). In addition, these findings are also consistent with an earlier study that found that systemic CL 316243 produced elevations of UCP-1 in mice (Yoshitomi et al., 1998).

While the combination treatment produced promising effects on both body weight and adiposity in Study 3, we acknowledge that one limitation to the study was the group size. This may have made it more difficult to measure more subtle changes in thermogenic gene expression and 4V OT-elicited changes in metabolic measures such as food intake and fat mass. We also did not anticipate that chronic 4V OT would produce such prolonged effects on T_*IBAT*_ in Study 2 animals yet fail to produce such an effect on T_*IBAT*_ in Study 3 animals. It is possible that this may be due, in part, to OT having had what appears to be a more delayed and less robust effect in Study 3 animals. We have previously shown that chronic third ventricular (3V) infusions of OT stimulate T_*IBAT*_ during a time that coincides with OT-elicited weight loss (days 2–3 of infusion period) (Roberts et al., 2017) and that OT appeared to maintain T_*IBAT*_ to that of control animals for the remainder of the infusion period (unpublished findings). In addition, following minipump removal and throughout the 4-week washout period, T_*IBAT*_ appeared to be slightly lower in rats that had been previously treated with chronic 3V OT relative to vehicle treated control rats (unpublished findings). Thus, OT may also function, in part, to help maintain weight loss by preventing a drop in BAT thermogenesis and energy expenditure (Blevins et al., 2016) that accompanies prolonged reductions of food intake and weight loss in animals (Fosgerau et al., 2014) and humans (Rosenbaum et al., 2002, 2005, 2010; Schwartz and Doucet, 2010). Current studies are underway to determine the extent to which SNS innervation of BAT is required for OT to increase energy expenditure and elicit weight loss.

In summary, our findings demonstrate that hindbrain OT administration in combination with systemic CL 316243 treatment results in more robust body weight loss relative to either treatment alone. In addition, the combination of OT and CL 316243 resulted in increased BAT thermogenesis based as determined by elevations of T_*IBAT*_, IBAT UCP-1 content and IBAT thermogenic gene expression. Collectively, our findings support the hypothesis that the combined treatment of OT and the beta-3 agonist, CL 316243, produces an additive effect to decrease body weight. The effects of the combined treatment on energy intake, fat mass, adipocyte size, and browning of EWAT were not additive and appear to be driven, in part, by transient changes in energy intake in response to either OT or CL 316243 alone while the effects on fat mass, adipocyte size, and browning of EWAT appear to be driven by CL 316243.

Collectively, these findings generate support for the use of OT either alone or as an adjunct to other weight loss therapies. Given that intranasal administration of OT can be an effective delivery approach to target the CNS (Striepens et al., 2013; Freeman et al., 2016; Smith et al., 2019) and to reduce food intake or elicit weight loss in lean or obese rodents (Maejima et al., 2015; Seelke et al., 2018), obese non-human primates (Blevins et al., 2015) and overweight and obese humans (Zhang et al., 2013; Lawson et al., 2015; Thienel et al., 2016; Hsu et al., 2017), future studies should address the extent to which intranasal treatment or chronic systemic infusions of OT alone and in combination with CL 316243 reduces body weight by reducing energy intake and/or increasing BAT thermogenesis or browning of WAT and which receptor populations contribute to these effects. Given the positive findings in male DIO rats and the promising effects of OT to reduce food intake and body weight in female rats (Liu et al., 2020) and female DIO mice (Maejima et al., 2017), respectively, future studies should also address the extent to which this combination treatment produces an additive effect to decrease body weight and adiposity in female DIO rodents.

## Data Availability Statement

The original contributions presented in the study are included in the article/Supplementary Material, further inquiries can be directed to the corresponding author/s.

## Ethics Statement

The animal study was reviewed and approved by Institutional Animal Care and Use Committee of the Veterans Affairs Puget Sound Health Care System (VAPSHCS) and the University of Washington.

## Author Contributions

JB conceived and designed the research and prepared the figures. ME, HN, AH, AD, MH, JS, TW, TW-H, JG, and JB performed the experiments. TW, MH, JS, TW-H, JG, and JB analyzed the data and interpreted results of experiments. ME, HN, TW, JG, PH, and JB drafted the manuscript. ME, HN, AH, AD, MH, JS, TW, TW-H, JG, KO’B, PH, and JB edited and revised the manuscript and approved the final version of manuscript. All authors contributed to the article and approved the submitted version.

## Conflict of Interest

JB has a financial interest in OXT Therapeutics, Inc., a company developing highly specific and stable analogs of oxytocin to treat obesity and metabolic disease. The remaining authors’ interests were reviewed and are managed by their local institutions in accordance with their conflict of interest policies and have nothing to report.

## Publisher’s Note

All claims expressed in this article are solely those of the authors and do not necessarily represent those of their affiliated organizations, or those of the publisher, the editors and the reviewers. Any product that may be evaluated in this article, or claim that may be made by its manufacturer, is not guaranteed or endorsed by the publisher.

## Supplementary Material

Supplementary Figure 1 Study paradigms. **(A)** Study 1, **(B)** Study 2, and **(C)** Study 3.

Supplementary Figure 2 Negative controls for UCP-1 staining in IWAT, EWAT, and IBAT. Images were taken from fixed (4% PFA) paraffin embedded sections (5 μm) containing **(A)** IWAT, **(B)** EWAT and **(C)** IBAT in HFD-fed rats treated with 4V OT (16 nmol/day) in combination with IP CL 316243 (0.5 mg/kg). Normal rabbit serum (1:100) was used in place of UCP-1 antibody (1:100); **(A–C)** all visualized at 100× magnification.
