# Supplementary figures and images for: Effects of Combined Oxytocin and Beta-3 Receptor Agonist (CL 316243) Treatment on Body Weight and Adiposity in Male Diet-Induced Obese Rats
Source: Front Physiol. 2021 Sep 8;12:725912. doi: 10.3389/fphys.2021.725912 (PMC8457402; doi:10.3389/fphys.2021.725912)

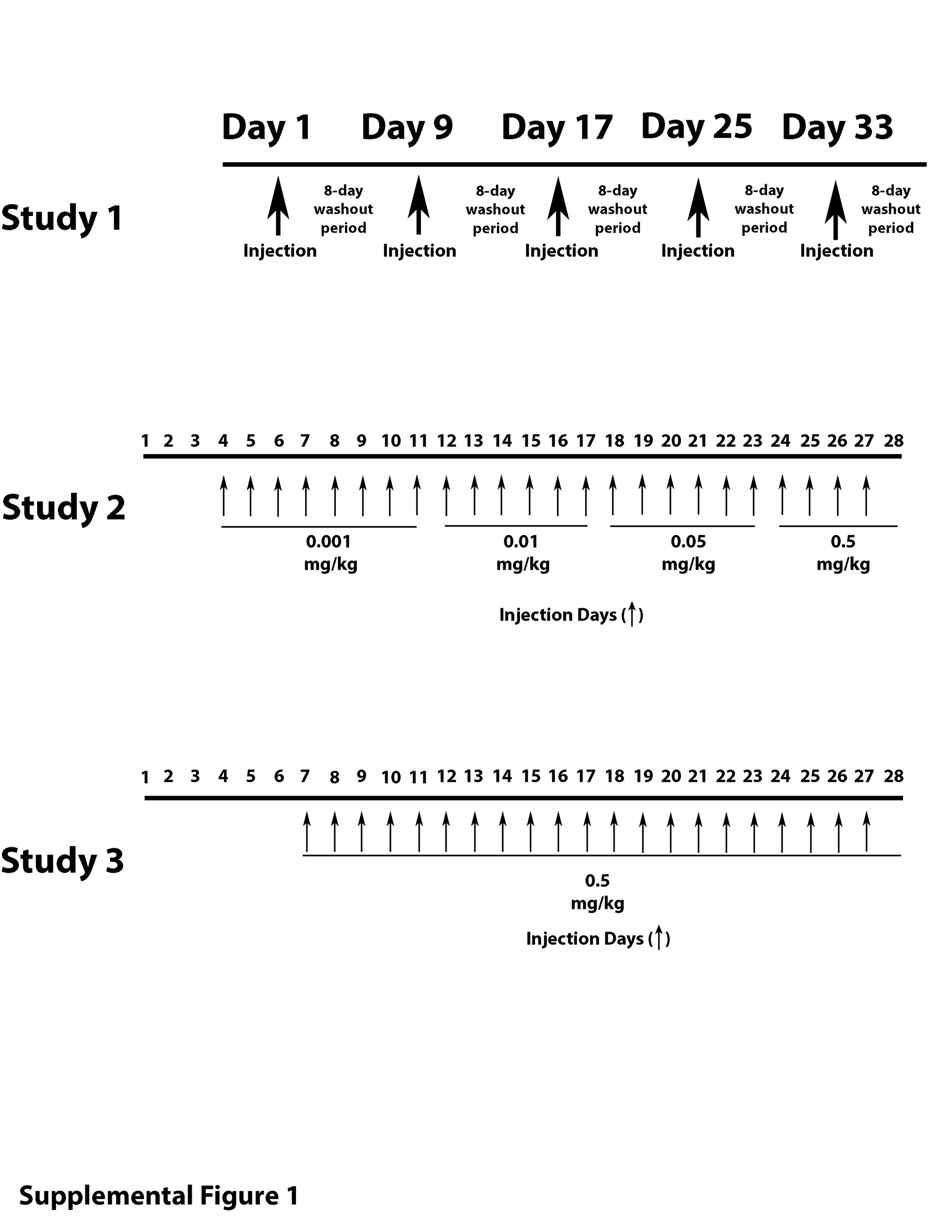

Supplement: Supplementary Figure 1 — Study paradigms. (A) Study 1, (B) Study 2, and (C) Study 3. [file Image_1.jpg]

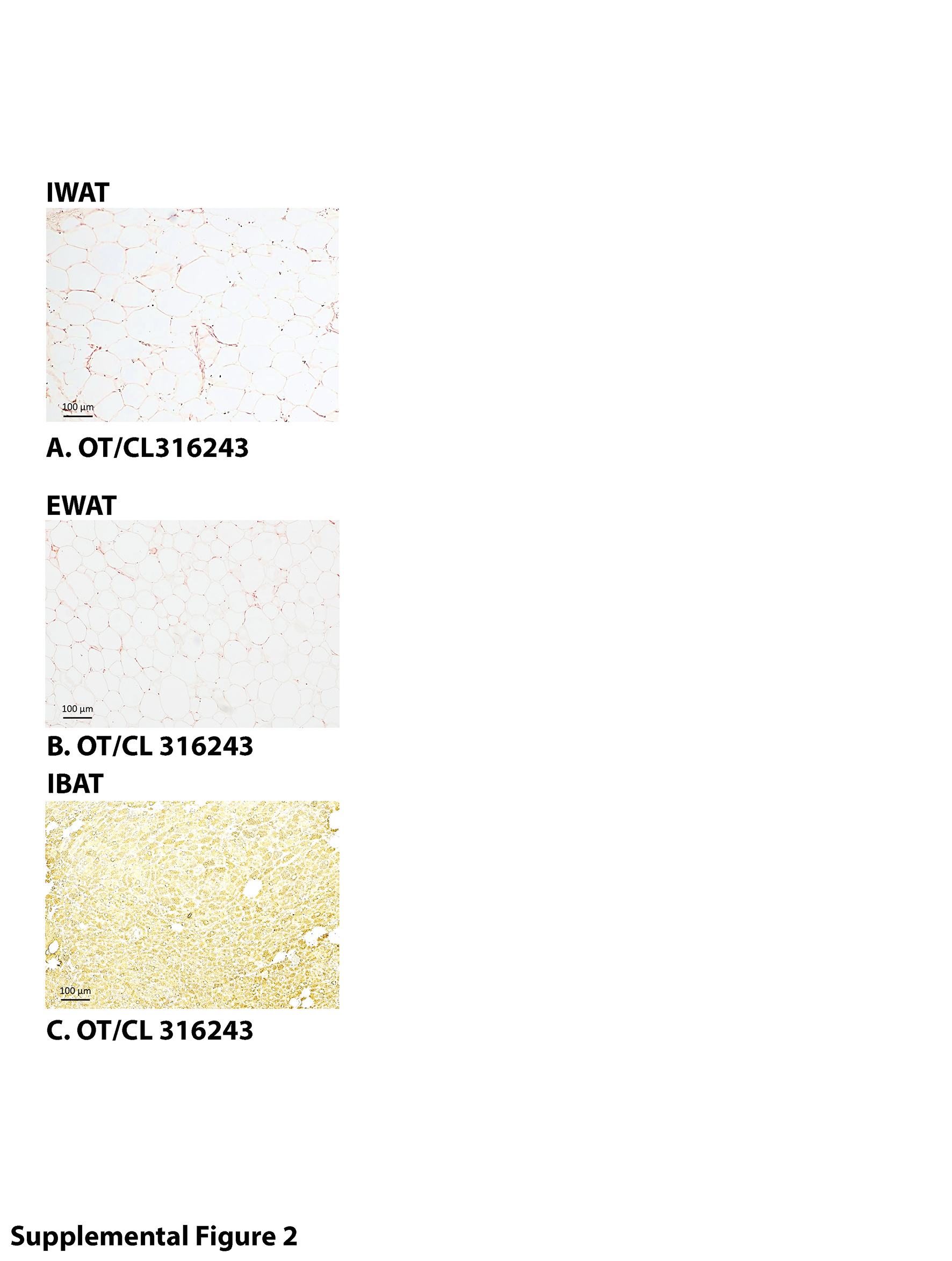

Supplement: Supplementary Figure 2 — Negative controls for UCP-1 staining in IWAT, EWAT, and IBAT. Images were taken from fixed (4% PFA) paraffin embedded sections (5 μm) containing (A) IWAT, (B) EWAT and (C) IBAT in HFD-fed rats treated with 4V OT (16 nmol/day) in combination with IP CL 316243 (0.5 mg/kg). Normal rabbit serum (1:100) was used in place of UCP-1 antibody (1:100); (A–C) all visualized at 100× magnification. [file Image_2.jpg]
